# The crystal structures and Hirshfeld surface analysis of three new bromo-substituted 3-methyl-1-(phenyl­sulfon­yl)-1*H*-indole derivatives

**DOI:** 10.1107/S2056989024004985

**Published:** 2024-05-31

**Authors:** S. Madhan, M. NizamMohideen, K. Harikrishnan, Arasambattu K. MohanaKrishnan

**Affiliations:** a Department of Physics, The New College, Chennai 600 014, University of Madras, Tamil Nadu, India; b Department of organic Chemistry, University of Madras, Guindy Campus, Chennai-600 025, Tamilnadu, India; National Taras Shevchenko University of Kyiv, Ukraine

**Keywords:** crystal structure, 1*H*-indole, phenyl­sulfonamide, π–π inter­actions, hydrogen bonding, Hirshfeld surface analysis

## Abstract

Three new 1*H*-indole derivatives, namely, 2-(bromo­meth­yl)-3-methyl-1-(phenyl­sulfon­yl)-1*H*-indole, C_16_H_14_BrNO_2_S, (**I**), 2-[(*E*)-2-(2-bromo-5-meth­oxy­phen­yl)ethen­yl]-3-methyl-1-(phenyl­sulfon­yl)-1*H*-indole, C_24_H_20_BrNO_3_S, (**II**), and 2-[(*E*)-2-(2-bromo­phen­yl)ethen­yl]-3-methyl-1-(phenyl­sulfon­yl)-1*H*-indole, C_23_H_18_BrNO_2_S, (**III**), exhibit nearly orthogonal orientations of their indole ring systems and sulfonyl-bound phenyl rings. Such conformations are favourable for inter­molecular bonding involving sets of slipped π–π inter­actions between the indole systems and mutual C—H⋯π hydrogen bonds, with the generation of two-dimensional monoperiodic patterns. The latter are found in all three structures, in the form of supra­molecular columns with every pair of successive mol­ecules related by inversion. The crystal packing of the compounds is additionally stabilized by weaker slipped π–π inter­actions between the outer phenyl rings (in **II** and **III**) and by weak C—H⋯O, C—H⋯Br and C—H⋯π hydrogen bonds. The structural significance of the different kinds of inter­actions agree with the results of a Hirshfeld surface analysis and the calculated inter­action energies. In particular, the largest inter­action energies (up to −60.8 kJ mol^−1^) are associated with pairing of anti­parallel indole systems, while the energetics of weak hydrogen bonds and phenyl π–π inter­actions are comparable and account for 13–34 kJ mol^−1^.

## Abstract 2

The crystal structures of three new indole derivative are described. The supra­molecular relations in the system were assessed with a Hirshfeld surface analysis and calculation of the inter­action energies, which suggest a primary significance of π–π and C—H⋯π inter­actions involving the indole moieties.

## 1. Chemical context

Derivatives of indole exhibit anti­bacterial (Okabe & Adachi, 1998 ▸) and anti­tumour (Schollmeyer *et al.*, 1995 ▸) activities. In particular, 1-(phenyl­sulfon­yl)indoles are applicable to the synthesis of biologically active alkaloids and their analogues, including pyridocarbazoles, such as the anti­cancer alkaloid ellipticine, carbazoles, furo­indoles, pyrrolo­indoles, indolocarbazoles and other species. Some of the phenyl­sulfonyl indole compounds have been shown to inhibit the HIV-1 RT enzyme in vitro and HTLVIIIb viral spread in MT-4 human T-lymphoid cells (Williams *et al.*, 1993 ▸). In such systems, the phenyl­sulfonyl moiety may act either as a protecting or an activating group (Jasinski *et al.*, 2009 ▸). Since the related halogen-substituted indoles also demonstrate anti­bacterial and anti­fungal activity (Piscopo *et al.*, 1990 ▸), one can anti­cipate a range of functional benefits from the halogen deriv­atization. Thus, substitution by bromine atoms may significantly enhance *in vitro* blood–brain barrier permeability, providing evidence for improved delivery to the central nervous system (Bouthenet *et al.*, 2011 ▸). Bromination on the phenol ring is important for the anti­microbial activity (Gentry *et al.*, 1999 ▸). The incorporation of heavy atoms, such as bromine, increases the generation of reactive species during photosensitization (Semenova *et al.*, 2021 ▸). In particular, fluorescent Br-substituted dyes are utilized for photodynamic therapy applications (Liu *et al.*, 2021 ▸). The fluorescent 4,6-di­bromo­indole­nine cyanine revealed excellent properties for optical tumour imaging (Guerrero *et al.*, 2017 ▸). Recognizing the importance of such compounds for biochemical applications and drug discovery and our ongoing research into the construction of indole derivatives have prompted us to investigate a series of Br-substituted species. We report herein the crystal structures determination and Hirshfeld surface analysis of three new indoles: 2-(bromo­meth­yl)-3-methyl-1-(phenyl­sulfon­yl)-1*H*-indole, C_16_H_14_BrNO_2_S, (**I**), 2-[(*E*)-2-(2-bromo-5-meth­oxy­phen­yl)ethen­yl]-3-methyl-1-(phenyl­sulfon­yl)-1*H*-indole, C_24_H_20_BrNO_3_S, (**II**), and 2-[(*E*)-2-(2-bromo­phen­yl)ethen­yl]-3-methyl-1-(phenyl­sulfon­yl)-1*H*-indole (**III**). 
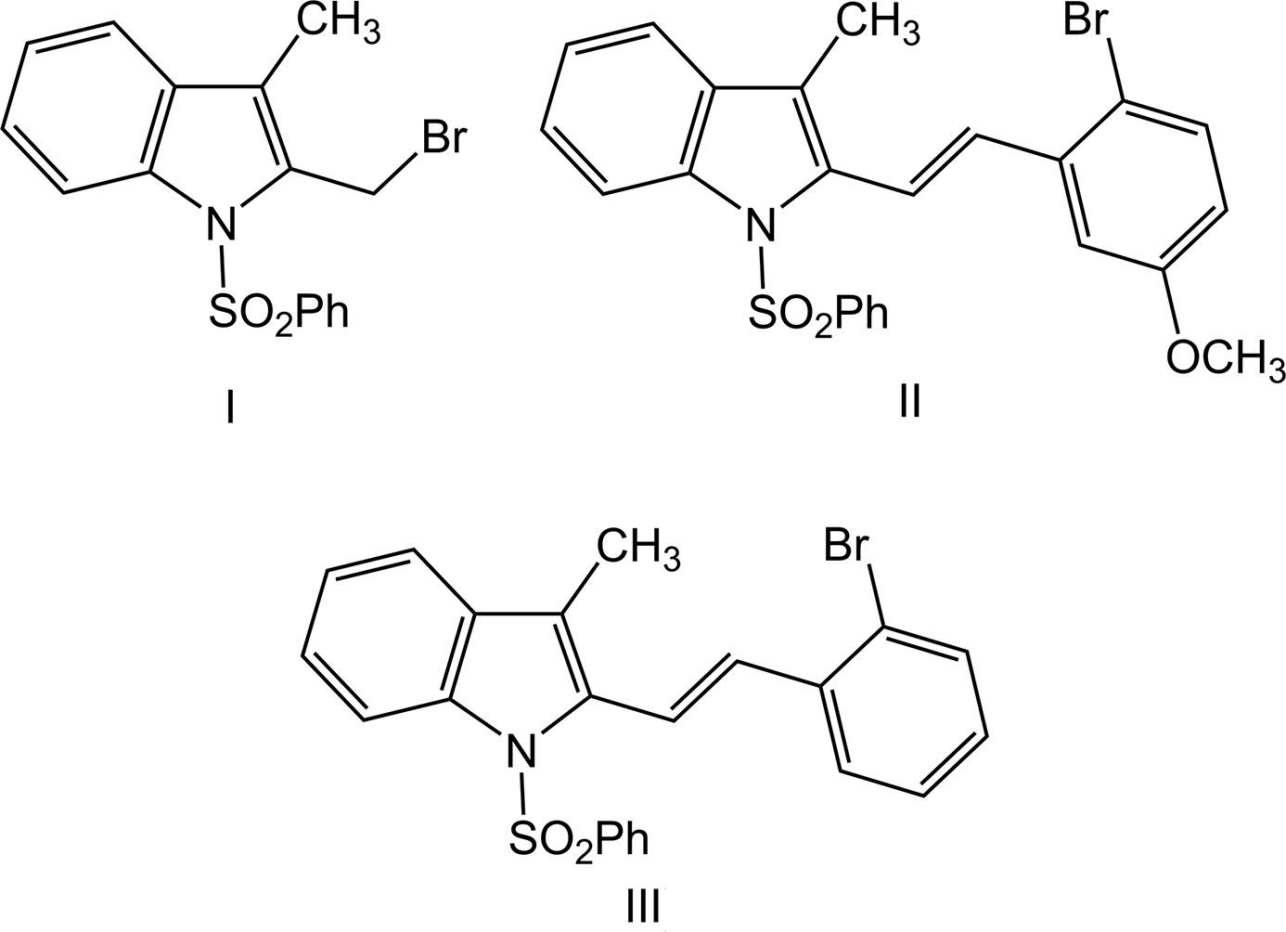


## 2. Structural commentary

**Figure 1:**
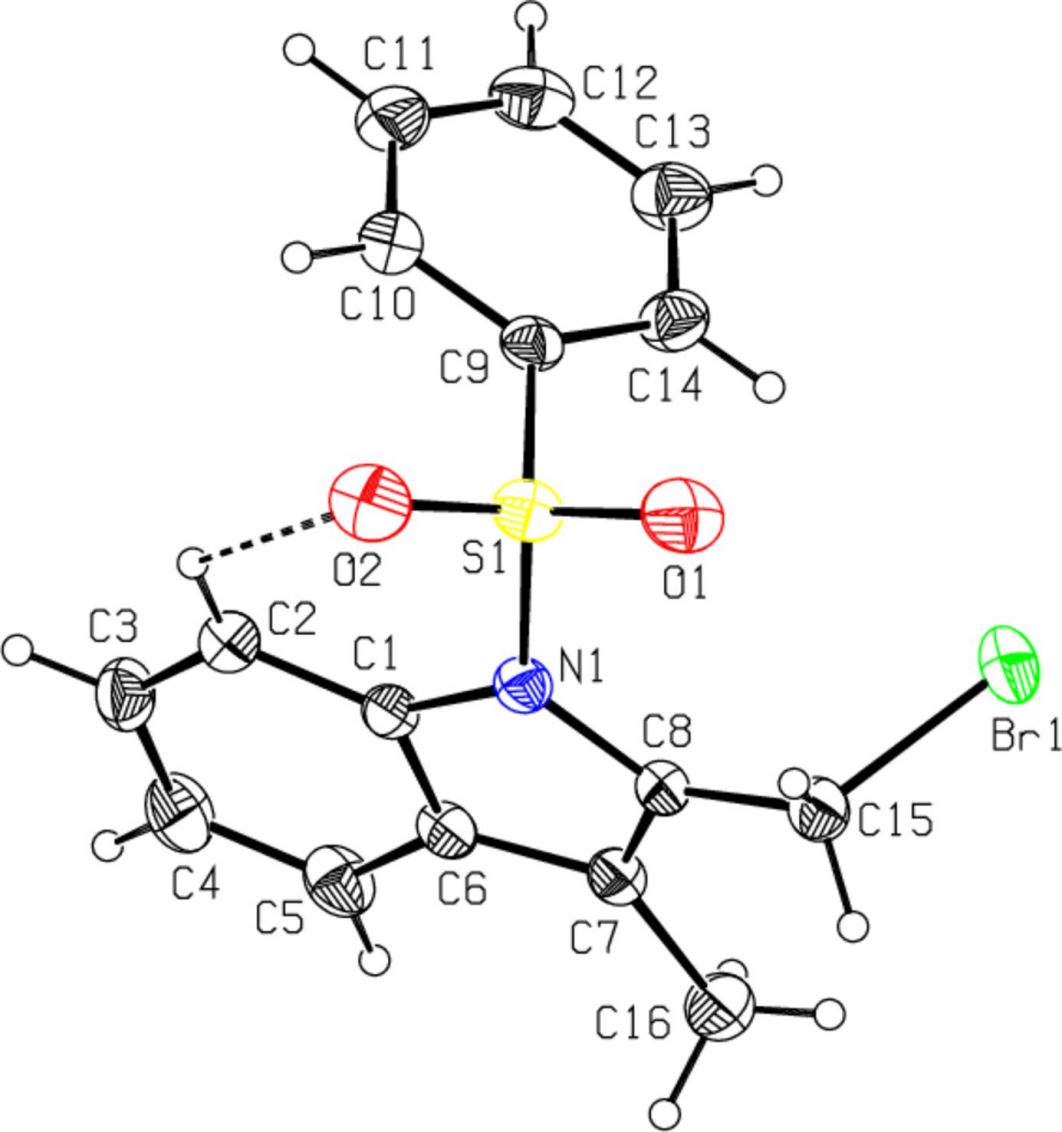
The mol­ecular structure of compound **I**, with atom labelling and displacement ellipsoids drawn at the 30% probability level. The dashed line indicates the intra­molecular hydrogen bond.

The mol­ecular structures of the title compounds, C_16_H_14_BrNO_2_S, (**I**), C_24_H_20_BrNO_3_S, (**II**) and C_23_H_18_BrNO_2_S, (**III**), are illustrated in Figs. 1 ▸, 2 ▸ and 3 ▸, respectively. In all the cases, the indole ring systems (N1/C1–C8) are essentially planar, with a maximum deviation from the corresponding mean plane of 0.0393 (17) Å, observed for N1 atom in **III**. The sulfonyl-bound phenyl rings (C9–C14) are almost orthogonal to the carrier indole ring systems (N1/C1–C8), with respective inter­planar angles of 76.40 (9)° for **I**, 73.35 (7)° for **II** and 87.68 (8)° for **III**. The ethenyl-bound phenyl rings (C17–C22) in **II** and **III** are also actually orthogonal to the indole frameworks, subtending dihedral angles of 72.48 (7) and 79.50 (8)°, respectively. As a consequence, the planes of these outer phenyl rings (C9–C14 and C17–C22) are nearly parallel, subtending angles of 9.56 (16) in **II** and 18.45 (6)° in **III**.

The torsion angles O2—S1—N1—C1 and O1—S1—N1—C8 [55.3 (2) and −21.1 (2)°, respectively, for **I**, −46.74 (19) and 45.94 (19)° for **II** and 42.9 (2) and −41.8 (2)° for **III**] indicate the *syn* conformation of the sulfonyl moiety. In all three compounds, the tetra­hedral configuration around S1 atom is somewhat distorted. The increase in the O2—S1—O1 angles [120.11 (14)° for **I**, 119.67 (12)° for **II** and 119.60 (13)° for **III**], with a simultaneous decrease in the N1—S1—C9 angles [104.46 (12)° for **I**, 103.78 (10)° for **II** and 105.70 (10)° for **III**] from the ideal tetra­hedral value (109.5°) are attributed to the Thorpe–Ingold effect (Bassindale, 1984 ▸). The widening of the angles may be due to the repulsive inter­action between the two short S=O bonds.

In all three compounds, the sum of the bond angles around N1 [355.88 (11), 348.62 (17) and 352.89 (12)° for **I**, **II** and **III**, respectively] indicate *sp* ^2^ hybridization (Beddoes *et al.*, 1986 ▸). At the same time, as a result of the electron-withdrawing character of the phenyl­sulfonyl groups, the N1—C*sp* ^2^ bonds are longer than the standard length value of 1.355 (14) Å [N1—C1 = 1.419 (3) for **I**, 1.425 (3) for **II** and 1.428 (3) Å for III and N1—C8 = 1.434 (3) for **I**, 1.438 (3) for **II** and 1.437 (3) Å for **III**] (Allen *et al.*, 1987 ▸; Cambridge Structural Database (CSD), Version 5.37; Groom *et al.*, 2016 ▸). In all the compounds, the certain expansion of the *ipso* angles at atoms C1, C3 and C4, and the contraction of the apical angles at atoms C2, C5 and C6 are caused by fusion of the smaller pyrrole ring with the six-membered benzene ring and the strain is taken up by the angular distortion rather than by bond-length distortion (Allen, 1981 ▸). The geometric parameters of the present compounds agree well with those reported for related structures (Madhan *et al.*, 2022 ▸, 2023*a* ▸,*b* ▸). In all three compounds, the mol­ecular conformations are stabilized by weak C2—H2⋯O2 intra­molecular inter­actions with C2⋯O2 = 2.950 (2)–3.057 (4) Å.

## 3. Supra­molecular features

With a lack of conventional hydrogen-bond donor functionality, the supra­molecular structures of all three compounds are dominated by weaker inter­actions, namely by weak C—H⋯O, C—H⋯Br and C—H⋯π hydrogen bonds (Tables 1 ▸–3 ▸ ▸) and slipped π–π stacking inter­actions (Table 4 ▸).

**Figure 4:**
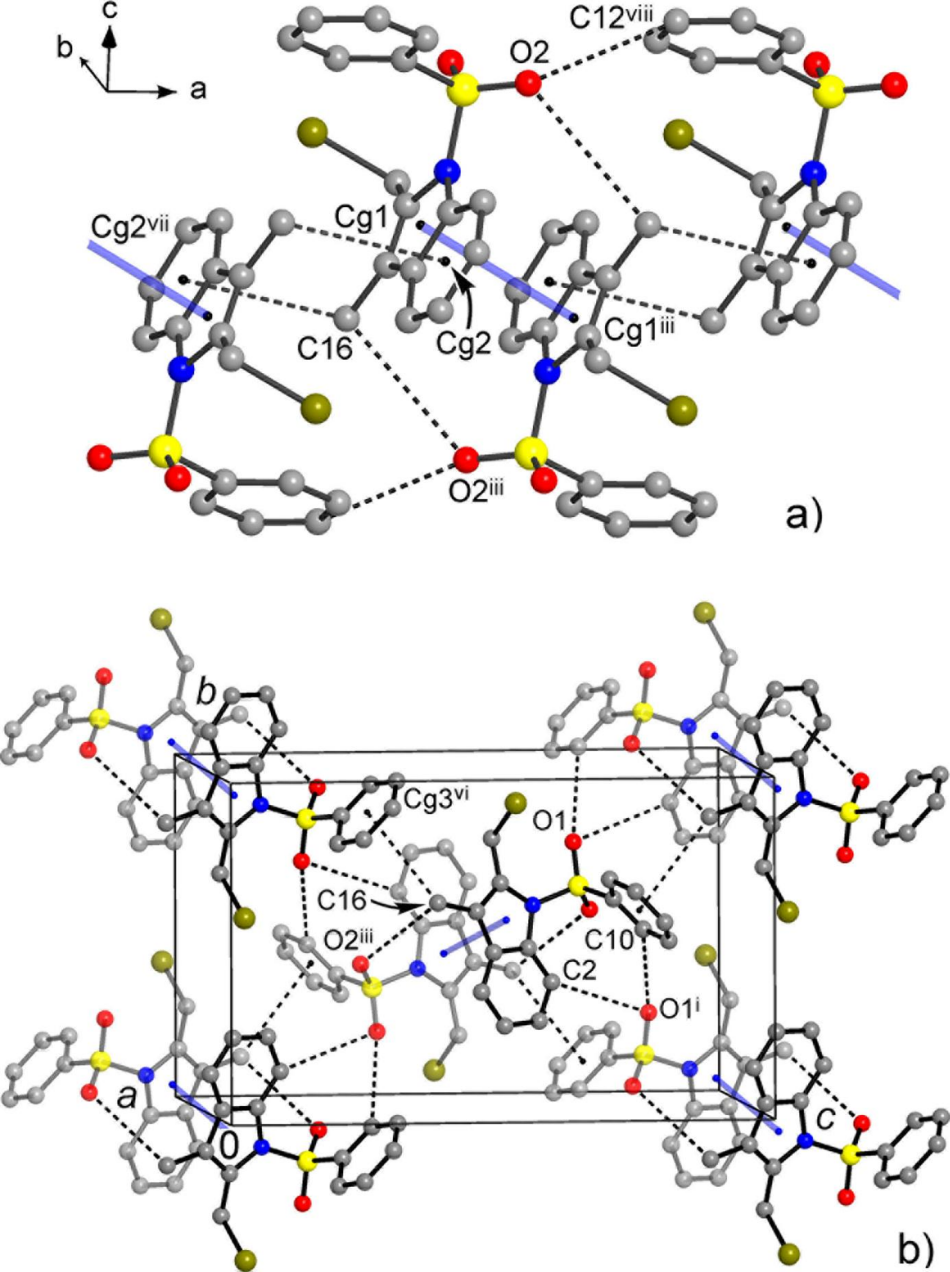
(*a*) Fragment of the structure of **I** showing a column of mol­ecules, down the *a*-axis in the crystal, sustained by π–π inter­actions and weak CH⋯π and CH⋯O bonds. (*b*) Projection of the structure nearly down the *a*-axis showing weak CH⋯O bonds between the columns (which are orthogonal to the drawing plane). Light-blue lines indicate the π–π inter­actions. [Symmetry codes: (i) *x* + 1, *y* − 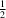 , −*z* + 1.5; (iii) *x* + 1, −*y* + 1, −*z* + 1; (vi) *x*, −*y* + 1.5, *z* − 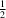 ; (vii) −*x*, −*y* + 1, −*z* + 1; (viii) *x* + 1, *y*, *z*.]

In the structure of **I**, mol­ecules are linked *via* double bonds involving C2—H2 and C10—H10 donors and O1^i^ acceptors [C⋯O = 3.306 (4) and 3.503 (4) Å; symmetry code: (i) −*x* + 1, *y* − 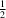 , −*z* + 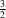 ] into the chains propagating along the *b*-axis direction in the crystal (Table 1 ▸). The most salient feature of the array is infinite stacking of the indole moieties, which yields columns down the *a*-axis. Within these columns, pairs of adjacent mol­ecules are held together by π–π inter­actions or by double CH_3_⋯π bonds, in alternate sequence (Fig. 4 ▸). The counterparts of every such pairs are related by inversion [symmetry codes: (iii) −*x* + 1, −*y* + 1, −*x* + 1; (v) −*x*, −*y* + 1, −*z* + 1, respectively.] For the dimer of the first kind, the geometry parameters are consistent with weak slipped π–π inter­actions. The shortest inter­centroid distance is observed between the pyrrole rings (Table 4 ▸). However, the centroid of the N1/C1–C8 group (*Cg*1) is situated almost above the midpoint of the C1 and C6 bridgehead atoms of the neighbouring mol­ecule and therefore both pyrrole–pyrrole [*Cg*1⋯*Cg*1^iii^ = 3.628 (3) Å] and pyrrole–benzo [*Cg*1⋯*Cg*2^iii^ = 3.831 (3) Å] inter­actions may be considered. The entire π–π and CH_3_⋯π bonded stack is additionally stabilized by weak hydrogen bonding of the sulfonyl O atoms [C⋯O = 3.302 (5)–3.702 (4) Å]. One can note the functional importance of the methyl group, which is a donor of three highly directional inter­actions, *viz*. the C—H⋯O bond and two C—H⋯π bonds (Table 1 ▸).

**Figure 5:**
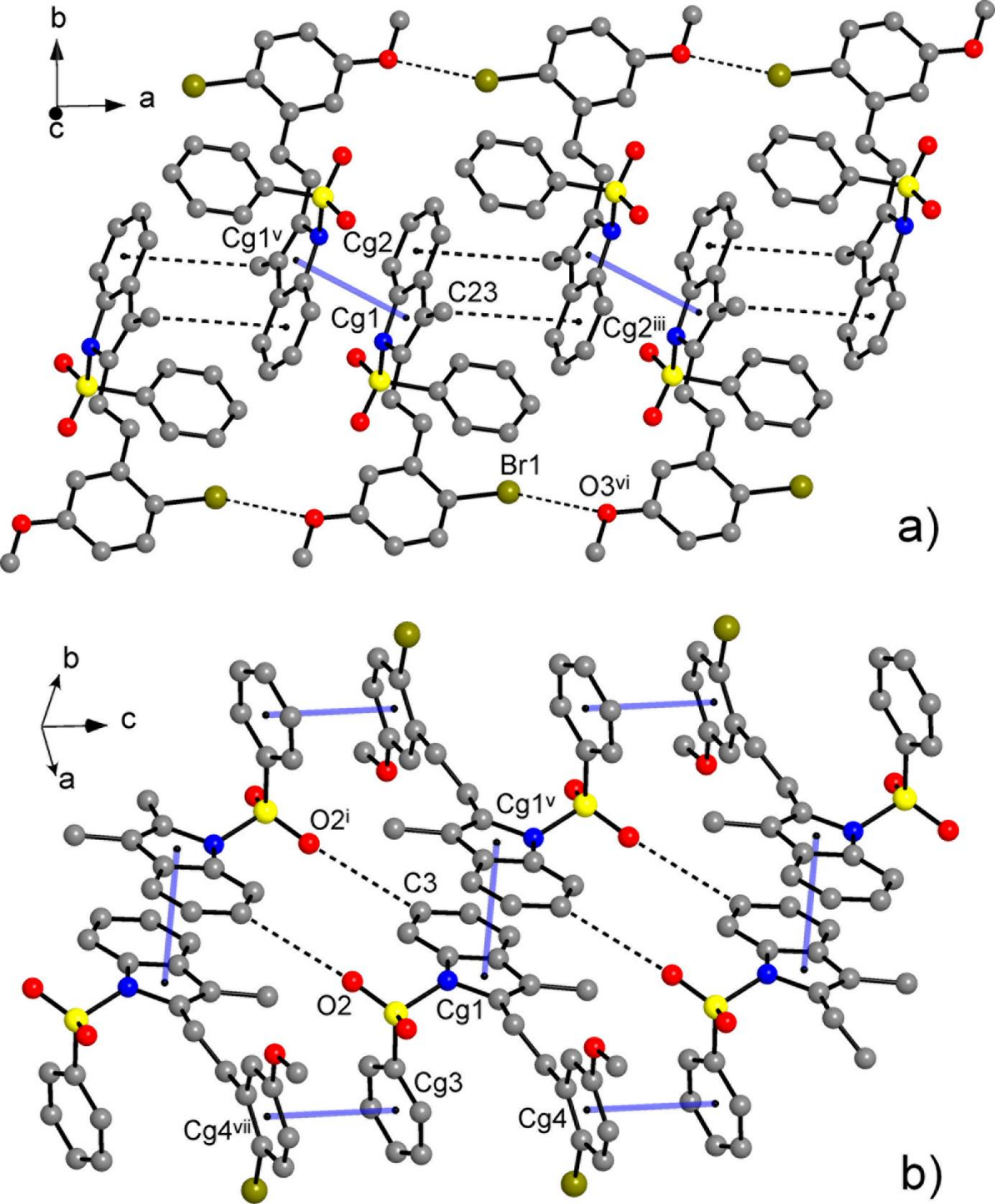
(*a*) Fragment of the structure of **II**, with columns of the mol­ecules down the *a*-axis, held by π–π (represented by light blue lines) and CH⋯π bonds. Short Br1⋯O3^vi^ contacts [3.3066 (19) Å] are also shown. (*b*) π–π and CH⋯O inter­actions between the columns. [Symmetry codes: (i) *x* + 1, −*y* + 1, −*z*; (iii) *x* + 2, −*y* + 1, −*z* + 1; (v) *x* + 1, −*y* + 1, −*z* + 1; (vi) *x* + 1, *y*, *z*; (vii) *x*, *y*, *z* − 1.]

The structure of **II** inherits the above motif (Fig. 5 ▸). In particular, a combination of π–π and CH_3_⋯π inter­actions assembles the mol­ecules into columns propagating along the *a*-axis direction in the crystal, in exactly the same manner as observed for compound **I**. In this case, the inter­actions are slightly weaker and the corresponding inter­centroid distances [*Cg*1⋯*Cg*1^v^ = 3.692 (3) Å; symmetry code: (v) −*x* + 1, −*y* + 1, −*z* + 1] are slightly larger compared with **II** (Table 4 ▸). The outer 2-bromo-5-meth­oxy­phenyl rings also contribute to the packing pattern since they afford π–π inter­actions with the sulfonyl-bound C9–C14 rings, with typical inter­centroid separations of 3.836 (2) Å and a relatively small slippage angle of 18.1 (2)° (Table 4 ▸). This stacking complements the weak C3—H3⋯O2^i^ hydrogen bonds [C⋯O = 3.448 (3) Å; symmetry code: (i) −*x* + 1, −*y* + 1, −*z*], linking the columns of mol­ecules in the *c*-axis direction (Fig. 5 ▸). There are no hydrogen-bonding inter­actions with the meth­oxy O3 atoms, which instead are involved in relatively short Br⋯O contacts of 3.3066 (19) Å. Very distal contacts of the type C24⋯*Cg*4^ix^ [4.098 (3) Å; *Cg*4 is the ring C17–C22 centroid; symmetry code: (ix) *x*, −*y* + 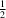 , *z* + 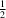 ] possibly indicate weak C—H⋯π inter­actions.

**Figure 6:**
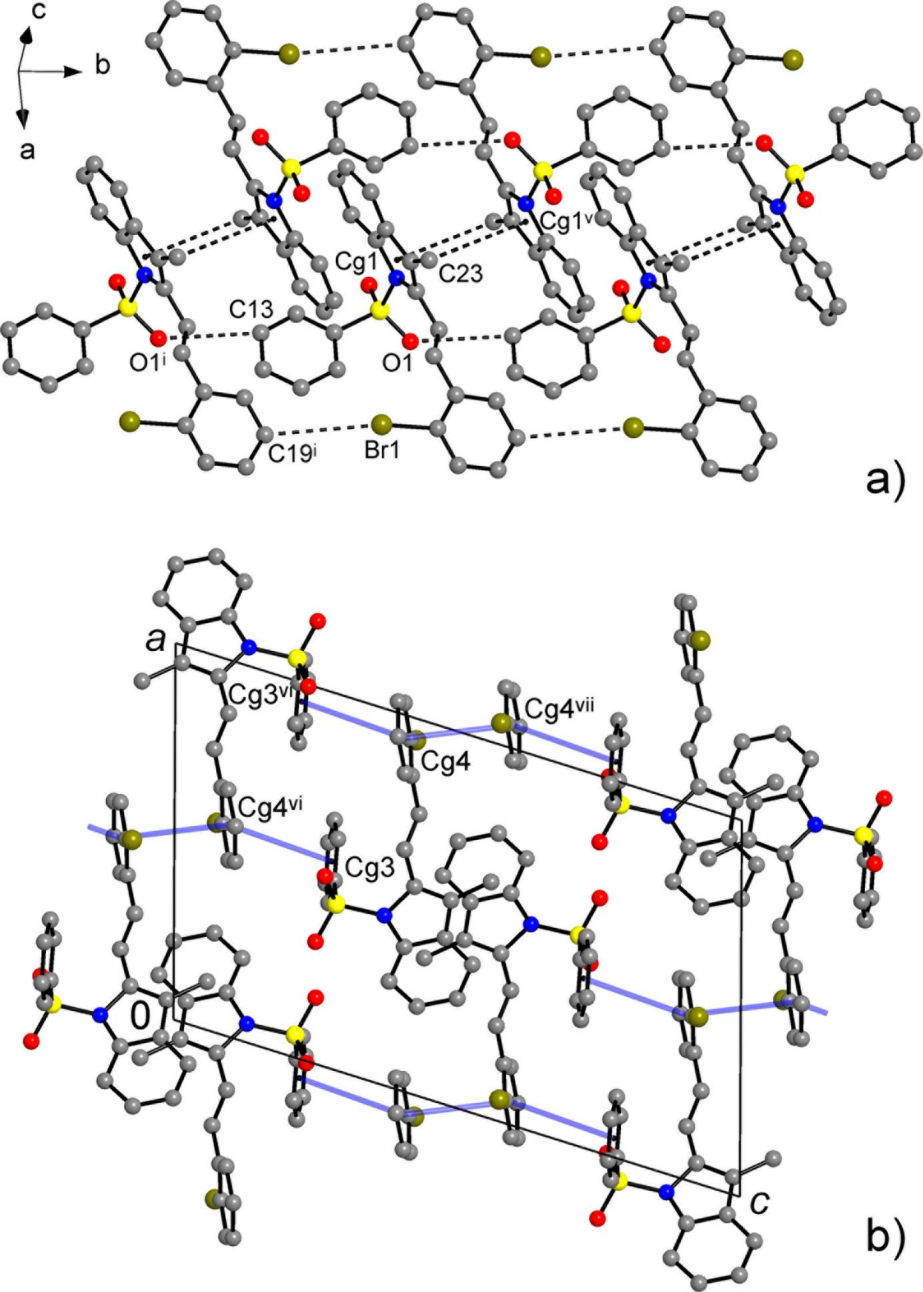
(*a*) Fragment of the structure of **III**, showing mutual CH⋯π bonding of the inversion-related indole fragments and how CH⋯O and CH⋯Br bonds contribute to the stabilization of the supra­molecular column. (*b*) Projection of the structure on the *ac*-plane. Note the extensive π–π inter­actions of the phenyl rings yielding four-decker sandwiches. The indole columns are orthogonal to the drawing plane. [Symmetry codes: (i) *x*, *y* − 1, *z*; (v) *x* + 1, −*y* + 2, −*z* + 1; (vi) *x* + 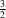 , *y* + 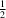 , −*z* + 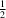 ; (vii) *x* + 2, −*y* + 2, −*z* + 1.]

In the structure of **III**, the π–π inter­actions of the indole ring systems are eliminated since the shortest inter­centroid distance exceeds 4.4 Å. However, the structure retains the double CH_3_⋯π bonding between inversion-related mol­ecules with C23⋯*Cg*1^v^ = 3.560 (3) Å [*Cg*1 is the centroid of the pyrrole ring N1/C1/C6–8; symmetry code: (v) −*x* + 1, −*y* + 2, −*z* + 1]. Moreover, these methyl groups also establish distal mutual contacts with the C1–C6 rings [C23⋯*Cg*2^iv^ = 3.999 (3) Å; symmetry code: (iv) −*x* + 1, −*y* + 1, −*z* + 1], which likely represent very weak CH_3_⋯π bonding. These inter­actions act in synergy with a set of weak C13—H13⋯O1^i^ and C19—H19⋯Br1^iii^ bonds (Table 3 ▸) to link the mol­ecules into the columns down the *b*-axis direction (Fig. 6 ▸). Therefore, the main features of the patterns seen for **I** and **II** are preserved for **III** with only minor variations. At the same time, beyond the supra­molecular columns, which are nearly intact for all three compounds, the bonding features for **III** are essentially different. Both kinds of the phenyl rings afford a set of π–π inter­actions with the generation of discrete tetra­mers (Fig. 6 ▸), with the central duo representing a stack of two anti­parallel inversion-related bromo­phenyl groups [*Cg*4⋯*Cg*4^vii^ = 3.691 (2) Å; symmetry code: (vii) −*x* + 2, −*y* + 2, −*z* + 1.] This central fragment is extended by incorporation of two outer sulfonyl-bound phenyl groups [*Cg*4⋯*Cg*3^vi^ = 3.742 (2) Å; symmetry code: (vi) −*x* + 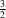 , *y* + 0.5, −*z* + 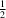 ].

## 4. Hirshfeld surface analysis

The supra­molecular inter­actions in the title structure were further assessed by Hirshfeld surface analysis. The Hirshfeld surfaces and 2D fingerprint plots were generated using *CrystalExplorer21* software (Spackman *et al.*, 2021 ▸).

**Figure 7:**
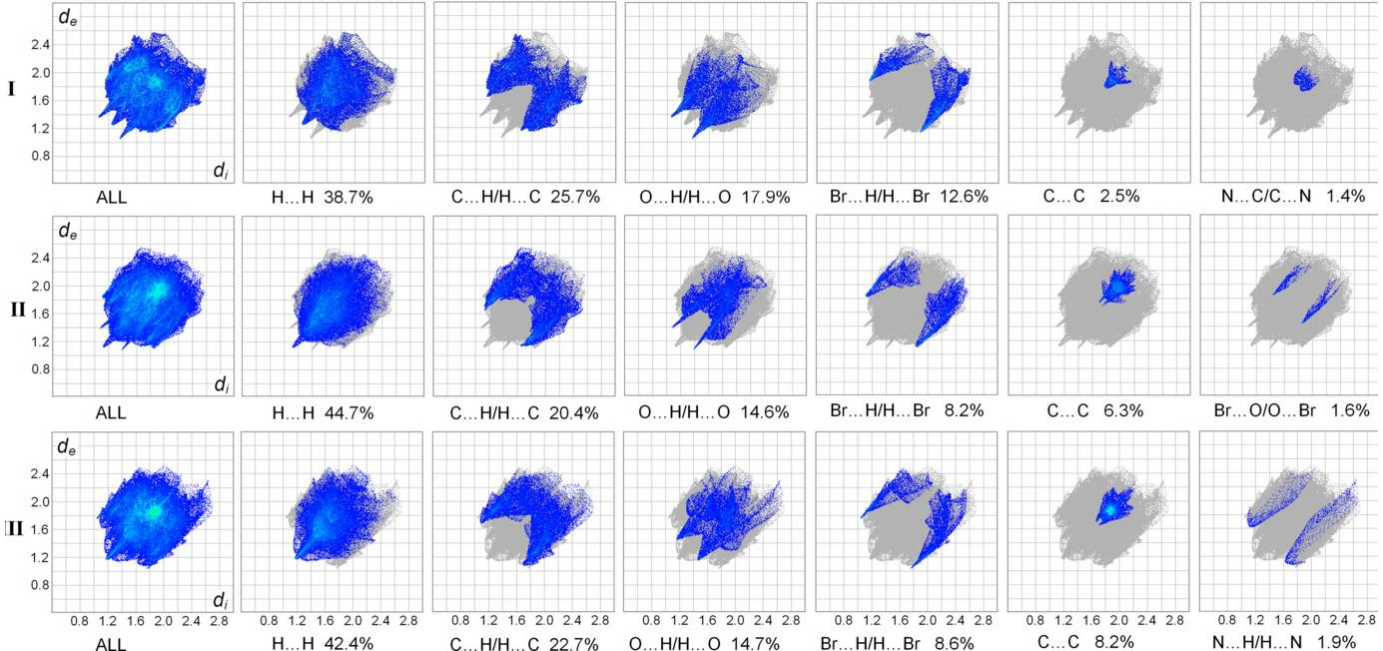
Two-dimensional fingerprint plots for **I**–**III** and delineated into the principal contributions of H⋯H, C⋯H/H⋯C, O⋯H/H⋯O, Br⋯H/H⋯Br, C⋯C, N⋯C/C⋯N, Br⋯O/O⋯Br and N⋯H/H⋯N contacts. Other contributors account for less than 1.0% contacts to the surface areas.

The two-dimensional fingerprint plots (Parkin *et al.*, 2007 ▸) detailing the various inter­actions for the mol­ecules are shown in Fig. 7 ▸. For all three compounds, Hirshfeld surfaces suggest the dominance of contacts with the hydrogen atoms, accounting for over 90% of the contacts. Beyond the largest fractions of H⋯H contacts (38.7–44.7%), the principal contributors are C⋯H/H⋯C (20.4–25.7%), O⋯H/H⋯O (14.6–17.9%) and Br⋯H/H⋯Br (8.2–12.6%) contacts corresponding to the different kinds of C—H⋯π, C—H⋯O or C—H⋯Br bonds. Every type of such bonding is readily identified by the plots representing pairs of diffuse spikes pointing to the lower left. One can note a common trend for suppression of such hydrogen bonding in **II** and **III**. For example, the contribution of the O⋯H/H⋯O contacts for **I** (17.9%) is perceptibly larger than for **II** (14.6%), which incorporates an additional meth­oxy O atom. This effect may be attributed to the increasing significance of π–π inter­actions for the crystal packing in the case of **II** and **III**, in line with the increased number of aromatic groups. In addition, a slight reduction in the Br⋯H/H⋯Br contacts (12.6% for **I** *versus* 8.2% and 8.6% for **II** and **III**, respectively) may be reflective of a weaker acceptor ability of the phenyl-bound Br atoms with respect to the bromo­methyl moieties in **I**. An overlap between nearly parallel aromatic frames, due to the slipped π–π stacking, is clearly indicated by the C⋯C plots for all compounds, in the form of the blue–green area centred at *ca d_e_* = *d_i_* = 1.85 Å. The plots suggest a progressive growth of the significance of these inter­actions, when moving from **I** to **II** and **III**. In line with this, the contributions of the C⋯C contacts to the entire surfaces are 2.5%, 6.3% and 8.2%, respectively. In the case of **II**, the peculiar short Br⋯O contacts are also readily identified by the fingerprint plots and they contribute as much as 1.6% to the surface area (Fig. 7 ▸).

**Figure 8:**
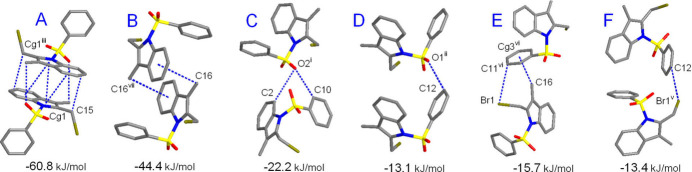
The principal pathways of the inter­molecular inter­actions for **I** representing π–π and weak hydrogen bonding, with a cut-off limit for calculated energies of 6.0 kJ mol^−1^. [Symmetry codes: (i) *x* + 1, *y* − 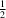 , −*z* + 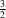 ; (ii) *x* − 1, −*y* + 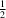 , *z* + 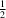 ; (iii) *x* + 1, −*y* + 1, −*z* + 1; (v) *x*, *y* − 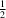 , −*z* + 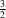 ; (vi) *x*, −*y* + 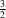 , *z* − 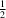 .]

**Figure 9:**
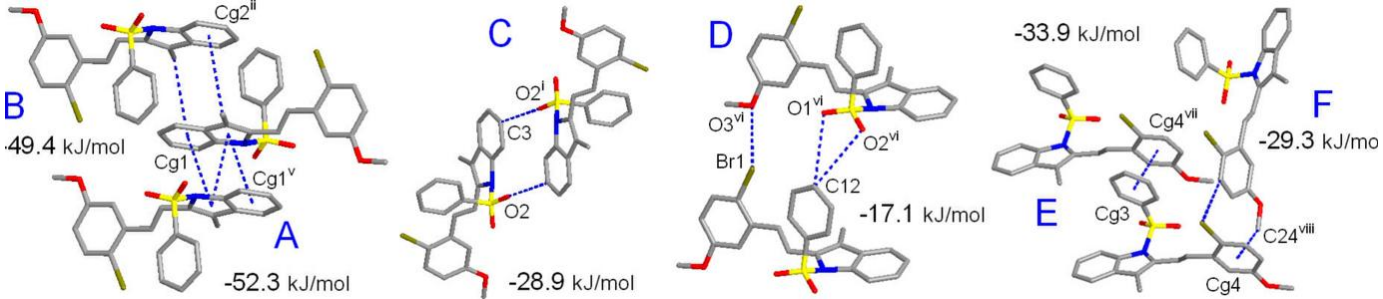
The principal pathways of the inter­molecular inter­actions for **II** representing π–π and weak hydrogen bonding, with a cut-off limit for calculated energies of 12.0 kJ mol^−1^. [Symmetry codes: (i) *x* + 1, −*y* + 1, −*z*; (iii) *x* + 2, −*y* + 1, −*z* + 1; (v) *x* + 1, −*y* + 1, −*z* + 1; (vi) *x* + 1, *y*, *z*; (vii) *x*, *y*, *z* − 1; (viii) *x*, −*y* + 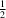 , *z* − 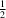 .]

The inter­action energy between the mol­ecules is expressed in terms of four components: electrostatic, polarization, dispersion and exchange repulsion. These energies were obtained using monomer wavefunctions calculated at the B3LYP/6-31G(d,p) level. The total inter­action energy, which is the sum of scaled components, was calculated for a 3.8 Å radius cluster of mol­ecules around the selected mol­ecule. The scale factors used in the CE-B3LYP bench research marked energy model (Mackenzie *et al.*, 2017 ▸) are given in Table 5 ▸. The principal inter­action pathways for **I**–**III** are shown in Figs. 8 ▸–10 ▸ ▸, respectively. The inter­action energies calculated by the energy model reveal that the inter­actions in the crystal have a significant contribution from dispersion components. It is worth noting that the primary forces for the crystal packing are associated with different stackings of the indole moieties. Either π–π or double CH⋯π inter­actions of the inversion-related mol­ecules are equally important and they are particularly prevalent in the case of **I**. Thus, the highest energy *E*_tot_ = −60.8 kJ mol^−1^ corresponds to the pairing pattern of type A (Fig. 8 ▸), with contributions from slipped π–π inter­actions and double C—H⋯O hydrogen bonding. In addition, short contacts of the methyl­ene groups C15 and C3—C4 bonds [C15⋯*Cg*(C3/C4)^iii^ = 3.412 (2) Å; symmetry code: (iii) −*x* + 1, −*y* + 1, −*z* + 1] possibly reflect a kind of weak tetrel C⋯π bonding. The energies of other types of indole/indole inter­actions for **II** and **III** are comparable [*E*_tot_ = −43.1 to −55.1 kJ mol^−1^] and the primary contributor here is London dispersion [up to −78.4 kJ mol^−1^], in accordance with the very large inter­action areas. The energies of the slipped π–π inter­actions of the phenyl rings in **II** and **III** are very similar and they account for −28.9 to −33.9 kJ mol^−1^ (Table 4 ▸). The significance of these inter­actions is comparable with weak C—H⋯O hydrogen bonds. The energies of the latter themselves are only medium, for example −13.1 kJ mol^−1^ (Type D) in **I** and −15.7 kJ mol^−1^ (Type F) in **II**. However, pairing of the mol­ecules *via* multiple hydrogen bonding increases the inter­action energies up to −28.9 kJ mol^−1^ (Type C in **II**, Fig. 9 ▸). This rich landscape of bonding modes, with a specific hierarchy of inter­action energies, could be applicable as a model for supra­molecular inter­actions of phenyl­sulfonyl-substituted indoles and their targeting of biomedical substrates.

## 5. Database survey

A search of the Cambridge Structural Database (Version 5.37; Groom *et al.*, 2016 ▸). indicated 123 compounds incorporating a phenyl­sulfonyl-1*H*-indole moiety. Of these compounds, several similar structures have been reported earlier, *i.e*. ethyl 2-acet­oxy­methyl-1-phenyl­sulfonyl-1*H*-indole-3-carboxyl­ate (Gunasekaran *et al.*, 2009 ▸), 3-iodo-2-methyl-1-phenyl­sulfonyl-1*H*-indole (Ramathilagam *et al.*, 2011 ▸) and 1-(2-bromo­methyl-1-phenyl­sulfonyl-1*H*-indol-3-yl)propan-1-one (Umadevi *et al.*, 2013 ▸). In these structures, the sulfonyl-bound phenyl rings are almost orthogonal to the indole ring systems, with corresponding dihedral angles of 83.35 (5), 82.84 (9) and 89.91 (11)°, respectively, being comparable with those in the present three compounds.

## 6. Synthesis and crystallization

Compound **I**: To a mixture of *N*-phenyl­sulfonyl-3-methyl­indole (6.00 g, 22.22 mmol) and paraformaldehyde (3.33 g, 111.1 mmol) in 50 ml of dry CCl_4_, a 33 wt % solution HBr in acetic acid (13.46 ml) was added rapidly. The mixture was kept at room temperature for 6 h. After completion of the reaction (monitored by TLC), the mixture was poured into 100 ml of ice–water and then extracted with CCl_4_ (2 × 20 ml). The extract was dried with Na_2_SO_4_. Removal of the solvent *in vacuo* followed by crystallization from methanol (4 ml) afforded compound **I** as a colourless solid (yield: 6.9 g, 86%).

Compound **II**: To a suspension of hexane (5 mL) washed NaH (0.43 g, 10.92 mmol) in dry THF (5 ml), a solution of diethyl {[3-methyl-1-(phenyl­sulfon­yl)-1*H*-indol-2-yl]meth­yl}phospho­nate (2.30 g, 5.46 mmol) in dry THF (10 ml) was slowly added *via* an addition funnel at 283 K under an N_2_ atmosphere and stirred for 15 min. Then a solution of 2-bromo-5-meth­oxy­benzaldehyde (1.39 g, 6.55 mmol) in dry THF (5 ml) was added and the mixture was allowed to stir for an additional 1 h. After completion of the reaction (monitored by TLC), the mixture was poured over crushed ice (100 g) containing concentrated HCl (1 ml). The solid formed was filtered and washed with methanol. Recrystallization from methanol (4 ml) afforded compound **II** as a bright-yellow solid (yield: 2.00 g, 76%). M.p. = 425–427 K.

Compound **III**: To a suspension of hexane (5 mL) washed NaH (0.38 g, 9.50 mmol) in dry THF (5 ml), a solution of diethyl {[3-methyl-1-(phenyl­sulfon­yl)-1*H*-indol-2-yl]meth­yl}phospho­nate (2.00 g, 4.75 mmol) in dry THF (10 ml) was slowly added *via* an addition funnel at 283 K under an N_2_ atmosphere and stirred for 15 min. Then a solution of 2-bromo­benzaldehyde (1.05 g, 5.70 mmol) in dry THF (5 ml) was added and the mixture was allowed to stir for an additional 1 h. After completion of the reaction (monitored by TLC), the mixture was poured over crushed ice (100 g) containing concentrated HCl (1 ml). The solid formed was filtered and washed with methanol to afford ethenyl­indole **III** as a bright-yellow solid (yield: 1.72 g, 71%). M.p. = 419-421 K.

## 7. Refinement

Crystal data, data collection and structure refinement details are summarized in Table 6 ▸. All C-bound H atoms were positioned geometrically and constrained to ride on their parent atoms with C—H = 0.93–0.97 Å with *U*_iso_(H) = 1.5*U*_eq_(C-meth­yl) and 1.2*U*_eq_(C) for other H atoms.

## Supplementary Material

Crystal structure: contains datablock(s) global, I, II, III. DOI: 10.1107/S2056989024004985/nu2005sup1.cif

Structure factors: contains datablock(s) I. DOI: 10.1107/S2056989024004985/nu2005Isup2.hkl

Structure factors: contains datablock(s) II. DOI: 10.1107/S2056989024004985/nu2005IIsup3.hkl

Structure factors: contains datablock(s) III. DOI: 10.1107/S2056989024004985/nu2005IIIsup4.hkl

Supporting information file. DOI: 10.1107/S2056989024004985/nu2005Isup5.cml

Supporting information file. DOI: 10.1107/S2056989024004985/nu2005IIsup6.cml

Supporting information file. DOI: 10.1107/S2056989024004985/nu2005IIIsup7.cml

CCDC references: 2358512, 2358511, 2358510

Additional supporting information:  crystallographic information; 3D view; checkCIF report

## Figures and Tables

**Figure 2:**
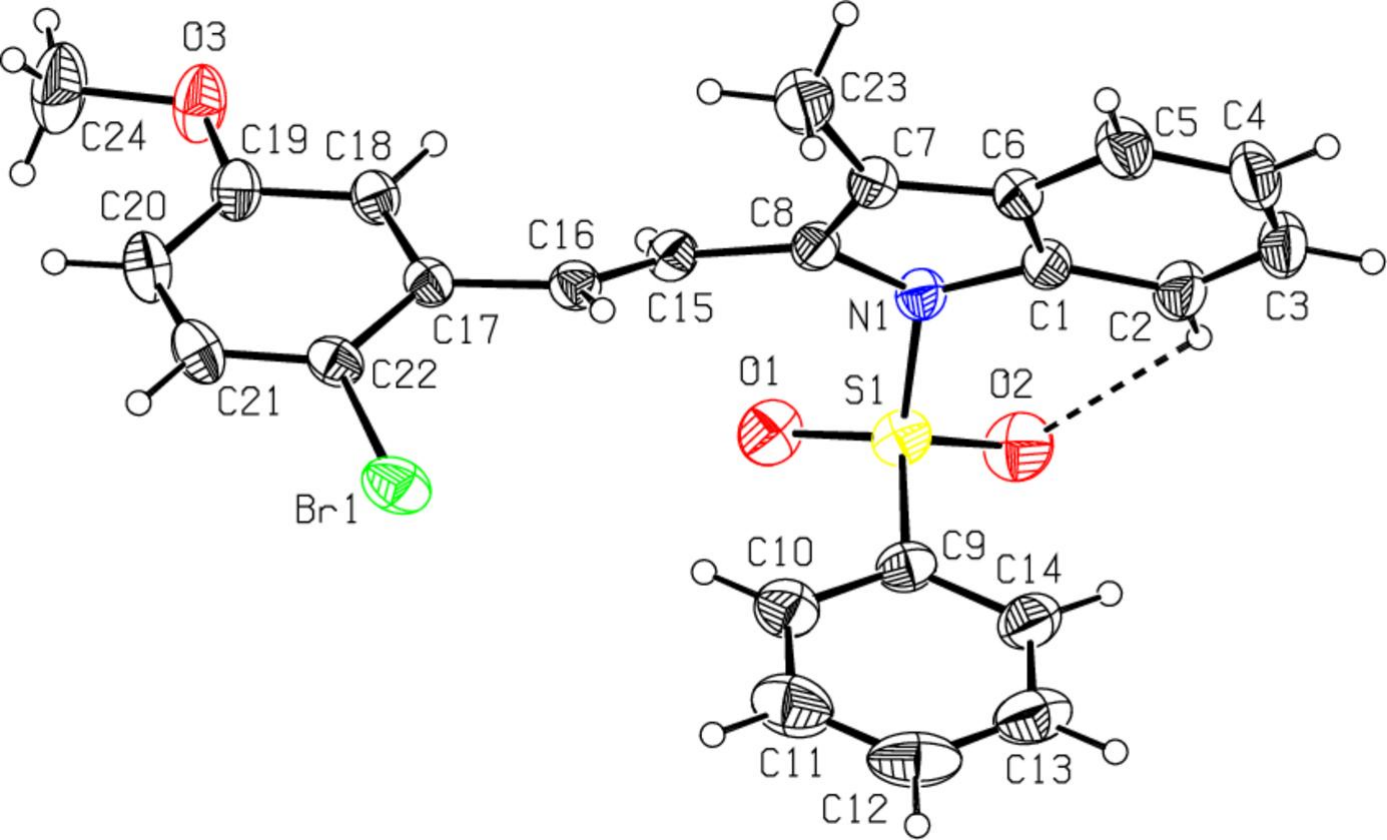
The mol­ecular structure of compound **II**, with atom labelling and displacement ellipsoids drawn at the 30% probability level. The dashed line indicates the intra­molecular hydrogen bond.

**Figure 3:**
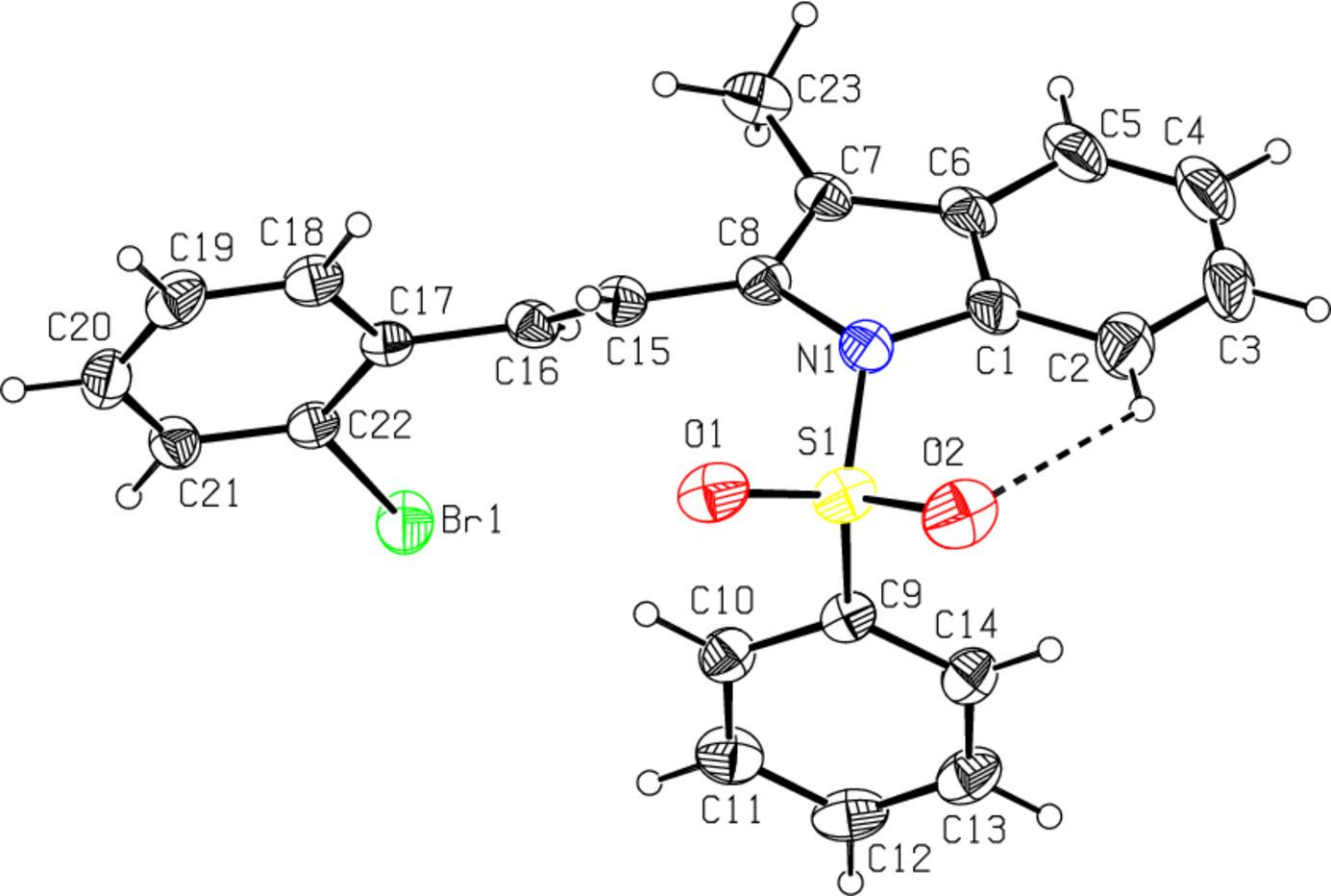
The mol­ecular structure of compound **III**, with atom labelling and displacement ellipsoids drawn at the 30% probability level. The dashed line indicates the intra­molecular hydrogen bond.

**Figure 10:**
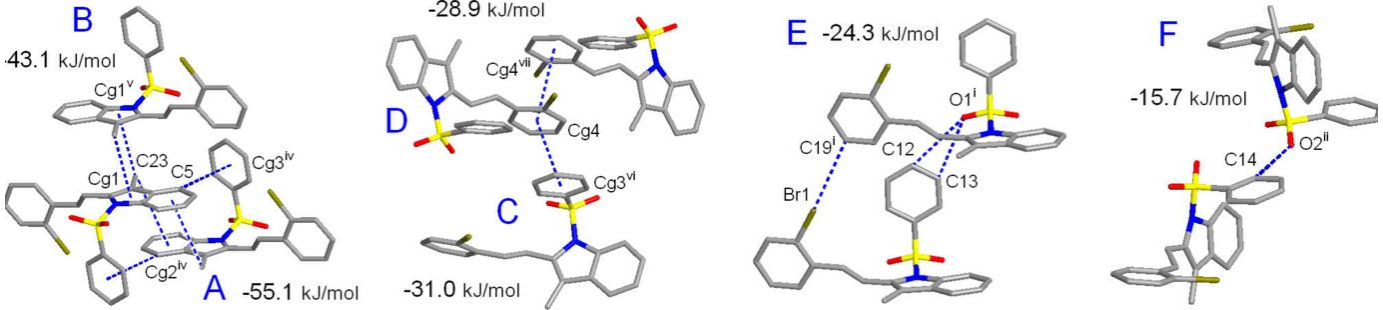
The principal pathways of the inter­molecular inter­actions for **II** representing π–π and weak hydrogen bonding, with a cut-off limit for calculated energies of 6.0 kJ mol^−1^. [Symmetry codes: (i) *x*, *y* − 1, *z*; (ii) *x* + 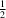 , *y* − 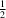 , −*z* + 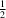 ; (iv) *x* + 1, −*y* + 1, −*z* + 1; (v) *x* + 1, −*y* + 2, −*z* + 1; (vi) *x* + 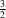 , *y* + 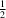 , −*z* + 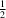 ; (vii) *x* + 2, −*y* + 2, −*z* + 1.]

**Table 1:** Hydrogen-bond geometry (Å, °) for **I** [Chem scheme1]

*D*—H⋯*A*	*D*—H	H⋯*A*	*D*⋯*A*	*D*—H⋯*A*
C2—H2⋯O2	0.93	2.49	3.057 (4)	120
C2—H2⋯O1^i^	0.93	2.71	3.503 (4)	144
C10—H10⋯O1^i^	0.93	2.53	3.306 (4)	141
C12—H12⋯O2^ii^	0.93	2.77	3.302 (5)	118
C13—H13⋯O2^ii^	0.93	2.92	3.376 (4)	112
C16—H16*C*⋯O2^iii^	0.96	2.76	3.702 (4)	168
C4—H4⋯Br1^iv^	0.93	3.16	3.905 (4)	138
C12—H12⋯Br1^v^	0.93	3.16	3.922 (4)	141
C14—H14⋯Br1	0.93	2.99	3.894 (4)	165
C16—H16*A*⋯*Cg*(C9–C14)^vi^	0.96	2.97	3.765 (4)	142
C16—H16*B*⋯*Cg*(C1–C6)^vii^	0.96	2.97	3.823 (5)	149

Symmetry codes: (i) 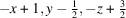 ; (ii) 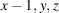 ; (iii) 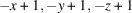 ; (iv) 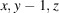 ; (v) 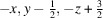 ; (vi) 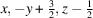 ; (vii) 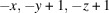 .

**Table 2:** Hydrogen-bond geometry (Å, °) for **II** [Chem scheme1]

*D*—H⋯*A*	*D*—H	H⋯*A*	*D*⋯*A*	*D*—H⋯*A*
C2—H2⋯O2	0.93	2.39	2.958 (4)	119
C3—H3⋯O2^i^	0.93	2.61	3.448 (3)	150
C24—H24*A*⋯Br1^ii^	0.96	3.03	3.699 (3)	128
C5—H5⋯*Cg*(C9–C14)^iii^	0.93	3.12	4.047 (3)	173
C20—H20⋯*Cg*(C17–C22)^iv^	0.93	3.15	3.978 (2)	149
C23—H23*C*⋯*Cg*(C1–C6)^iii^	0.96	3.11	4.036 (4)	162

Symmetry codes: (i) 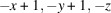 ; (ii) 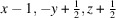 ; (iii) 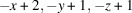 ; (iv) 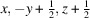 .

**Table 3:** Hydrogen-bond geometry (Å, °) for **III** [Chem scheme1]

*D*—H⋯*A*	*D*—H	H⋯*A*	*D*⋯*A*	*D*—H⋯*A*
C2—H2⋯O2	0.93	2.37	2.949 (5)	120
C13—H13⋯O1^i^	0.93	2.73	3.420 (3)	132
C14—H14⋯O2^ii^	0.93	2.77	3.547 (4)	142
C19—H19⋯Br1^iii^	0.93	2.94	3.805 (3)	155
C5—H5⋯*Cg*(C9–C14)^iv^	0.93	2.96	3.806 (4)	153
C23—H23*A*⋯*Cg*(C1–C6)^iv^	0.96	3.21	3.999 (3)	110
C23—H23*B*⋯*Cg*(N1/C1/C6–C8)^v^	0.96	3.12	3.561 (3)	149

Symmetry codes: (i) 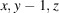 ; (ii) 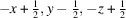 ; (iii) 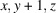 ; (iv) 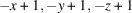 ; (v) 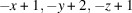 .

**Table 4:** Geometry of stacking inter­actions (Å, °) for **I**–**III** *Cg* is a group centroid; plane⋯*CgB* is the distance between the mean plane of Group *A* and the centroid of the inter­acting Group *B*; ipa is the inter­planar angle; sa is the slippage angle, which is the angle of the *CgA*⋯*CgB* axis to the Group *A* mean plane normal.

Compound	Group *A*	Group *B*	Shortest contacts	*CgA*⋯*CgB*	Plane⋯*CgB*	ipa	sa
**I**	(N1/C1–C8)	(N1/C1–C8)^iii^	3.573 (4)	3.628 (2)	3.551 (2)	0	11.8 (2)
	(N1/C1–C8)	(C1–C6)^iii^	3.573 (4)	3.831 (2)	3.552 (2)	0.16 (14)	22.0 (2)
**II**	(N1/C1–C8)	(N1/C1–C8)^v^	3.618 (3)	3.692 (2)	3.633 (2)	0	10.3 (2)
	(N1/C1–C8)	(C1–C6)^v^	3.618 (3)	3.975 (2)	3.635 (2)	0.62 (13)	23.9 (2)
	(C17–C22)	(C19–C14)^vii^	3.463 (3)	3.836 (3)	3.646 (3)	9.56 (16)	18.1 (2)
**III**	(C17–C22)	(C9–C14)^vi^	3.381 (3)	3.742 (2)	3.379 (2)	10.34 (7)	24.5 (2)
	(C17–C22)	(C17–C22)^vii^	3.489 (3)	3.691 (2)	3.488 (2)	0	19.1 (2)

Symmetry codes for **I**: (iii) −*x* + 1, −*y* + 1, −*z* + 1; for **II**: (v) −*x* + 1, −*y* + 1, −*z* + 1; (vii) *x*, *y*, *z* − 1; for **III**: (vi) −*x* + 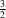 , *y* + 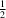 , −*z* + 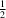 ; (vii) −*x* + 2, −*y* + 2, −*z* + 1.

**Table 5:** Calculated inter­action energies (kJ mol^−1^) for **I**–**III** Inter­action energies were calculated employing the CE-B3LYP/6–31G(d,p) functional/basis set combination. The scale factors used to determine *E*_tot_ were: *k*_ele_ = 1.057, *k*_pol_ = 0.740, *k*_dis_ = 0.871, and *k*_rep_ = 0.618 (Mackenzie *et al.*, 2017 ▸). For details of the inter­action modes, see Figs. 8 ▸–10 ▸ ▸; *R* is the distance in Å between the centroids of inter­acting mol­ecules.

Type	Symmetry code	Inter­action	*R*	*E*_ele_	*E*_pol_	*E*_dis_	*E*_rep_	*E*_tot_
Compound **I**								
*A*	−*x* + 1, −*y* + 1, −*z* + 1	π–π, CH⋯O	6.45	−20.2	−4.5	−71.5	42.4	−60.8
*B*	−*x*, −*y* + 1, −*z* + 1	CH–π	6.35	−10.5	−2.0	−59.9	32.8	−44.4
*C*	−*x* + 1, *y* − 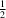 , −*z* + 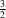	CH⋯O	7.79	−7.9	−3.6	−22.8	14.0	−22.2
*D*	*x* − 1, *y*, *z*	CH⋯O	7.98	−5.7	−2.0	−10.6	6.0	−13.1
*E*	*x*, −*y* + 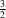 , *z* − 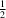	CH⋯Br, CH⋯π	9.09	−5.0	−0.9	−18.6	10.4	−15.7
*F*	−*x*, *y* − 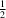 , −*z* + 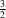	CH⋯Br	9.03	−2.3	−1.0	−17.8	8.5	−13.4
Compound **II**								
*A*	−*x* + 1, −*y* + 1, −*z* + 1	π–π	8.08	−8.9	−2.9	−67.3	29.0	−52.3
*B*	−*x* + 2, −*y* + 1, −*z* + 1	CH—π	8.10	−15.7	−1.7	−67.9	44.6	−49.4
*C*	−*x* + 1, −*y* + 1, −*z*	CH⋯O	12.70	−12.6	−2.9	−15.4	0.0	−28.9
*D*	*x* + 1, *y*, *z*	CH⋯O	8.43	−7.1	−2.2	−18.7	13.6	−17.1
*E*	*x*, *y*, *z* − 1	π–π	8.95	−4.2	−2.6	−47.8	22.8	−33.9
*F*	*x*, −*y* + 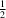 , *z* − 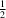	CH⋯Br, CH⋯π	8.77	−6.2	−2.2	−38.6	20.2	−29.3
Compound **III**								
*A*	−*x* + 1, −*y* + 1, −*z* + 1	CH⋯π, dispersion	7.64	−11.5	−1.9	−78.4	43.3	−55.1
*B*	−*x* + 1, −*y* + 2, −*z* + 1	CH⋯π	7.91	−9.3	−2.0	−57.5	29.5	−43.1
*C*	−*x* + 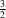 , *y* + 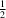 , −*z* + 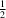	π–π	7.85	−7.1	−1.7	−40.7	21.3	−31.0
*D*	−*x* + 2, −*y* + 2, −*z* + 1	π–π	9.91	−5.2	−1.1	−50.4	34.5	−28.9
*E*	*x*, *y* − 1, *z*	CH⋯O, CH⋯Br	8.35	−10.5	−2.7	−28.3	21.7	−24.3
*F*	−*x* + 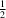 , *y* − 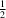 , −*z* + 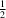	CH⋯O	10.52	−3.6	−2.7	−15.6	6.0	−15.7

**Table 6:** Experimental details

	**I**	**II**	**III**
Crystal data			
Chemical formula	C_16_H_14_BrNO_2_S	C_24_H_20_BrNO_3_S	C_23_H_18_BrNO_2_S
*M*_r_	364.25	482.38	452.35
Crystal system, space group	Monoclinic, *P*2_1_/*c*	Monoclinic, *P*2_1_/*c*	Monoclinic, *P*2_1_/*n*
Temperature (K)	298	298	298
*a*, *b*, *c* (Å)	7.979 (6), 11.100 (8), 17.540 (14)	8.4252 (9), 28.669 (3), 8.9462 (11)	12.5530 (8), 8.3533 (5), 19.7698 (11)
β (°)	99.04 (3)	95.445 (4)	107.078 (2)
*V* (Å^3^)	1534 (2)	2151.1 (4)	1981.6 (2)
*Z*	4	4	4
Radiation type	Mo *K*α	Mo *K*α	Mo *K*α
μ (mm^−1^)	2.82	2.03	2.20
Crystal size (mm)	0.30 × 0.24 × 0.07	0.25 × 0.20 × 0.13	0.36 × 0.31 × 0.24

Data collection			
Diffractometer	Bruker D8 Venture Diffractometer	Bruker D8 Venture Diffractometer	Bruker D8 Venture Diffractometer
Absorption correction	Multi-scan (*SADABS*; Krause *et al.*, 2015 ▸)	Multi-scan (*SADABS*; Krause *et al.*, 2015 ▸)	Multi-scan (*SADABS*; Krause *et al.*, 2015 ▸)
*T*_min_, *T*_max_	0.589, 0.753	0.555, 0.745	0.514, 0.745
No. of measured, independent and observed [*I* > 2σ(*I*)] reflections	66991, 4464, 3158	52479, 5296, 3905	50531, 4293, 3516
*R*_int_	0.049	0.077	0.058
(sin θ/λ)_max_ (Å^−1^)	0.704	0.666	0.639

Refinement			
*R*[*F* ^2^ > 2σ(*F* ^2^)], *wR*(*F* ^2^), *S*	0.046, 0.118, 1.10	0.039, 0.099, 1.03	0.036, 0.087, 1.10
No. of reflections	4464	5296	4293
No. of parameters	191	272	254
H-atom treatment	H-atom parameters constrained	H-atom parameters constrained	H-atom parameters constrained
Δρ_max_, Δρ_min_ (e Å^−3^)	0.75, −0.80	0.39, −0.68	0.37, −0.32

Computer programs: *APEX2* and *SAINT* (Bruker, 2016 ▸), *SHELXS2018/3* (Sheldrick, 2008 ▸), *SHELXL2018/3* (Sheldrick, 2015 ▸), *ORTEP-3 for Windows* and *WinGX* (Farrugia, 2012 ▸), *Mercury* (Macrae *et al.*, 2020 ▸), *publCIF* (Westrip, 2010 ▸) and *PLATON* (Spek, 2020 ▸).

## supplementary crystallographic information

### 2-(Bromomethyl)-3-methyl-1-(phenylsulfonyl)-1H-indole (I) Crystal data

**Table:**

C_16_H_14_BrNO_2_S	*F*(000) = 736
*M**_r_* = 364.25	*D*_x_ = 1.577 Mg m^−^^3^
Monoclinic, *P*2_1_/*c*	Mo *K*α radiation, λ = 0.71073 Å
*a* = 7.979 (6) Å	Cell parameters from 66991 reflections
*b* = 11.100 (8) Å	θ = 1.4–25.0°
*c* = 17.540 (14) Å	µ = 2.82 mm^−^^1^
β = 99.04 (3)°	*T* = 298 K
*V* = 1534 (2) Å^3^	Prism, colorless
*Z* = 4	0.30 × 0.24 × 0.07 mm

### 2-(Bromomethyl)-3-methyl-1-(phenylsulfonyl)-1H-indole (I) Data collection

**Table:**

Bruker D8 Venture Diffractometer	3158 reflections with *I* > 2σ(*I*)
Radiation source: micro focus sealed tube	*R*_int_ = 0.049
ω and φ scans	θ_max_ = 30.0°, θ_min_ = 3.2°
Absorption correction: multi-scan (*SADABS*; Krause *et al.*, 2015)	*h* = −11→10
*T*_min_ = 0.589, *T*_max_ = 0.753	*k* = −15→15
66991 measured reflections	*l* = −24→24
4464 independent reflections	

### 2-(Bromomethyl)-3-methyl-1-(phenylsulfonyl)-1H-indole (I) Refinement

**Table:**

Refinement on *F*^2^	Primary atom site location: structure-invariant direct methods
Least-squares matrix: full	Secondary atom site location: difference Fourier map
*R*[*F*^2^ > 2σ(*F*^2^)] = 0.046	Hydrogen site location: inferred from neighbouring sites
*wR*(*F*^2^) = 0.118	H-atom parameters constrained
*S* = 1.10	*w* = 1/[σ^2^(*F*_o_^2^) + (0.0434*P*)^2^ + 1.2291*P*] where *P* = (*F*_o_^2^ + 2*F*_c_^2^)/3
4464 reflections	(Δ/σ)_max_ = 0.001
191 parameters	Δρ_max_ = 0.75 e Å^−^^3^
0 restraints	Δρ_min_ = −0.80 e Å^−^^3^

### 2-(Bromomethyl)-3-methyl-1-(phenylsulfonyl)-1H-indole (I) Special details

**Table:**

Geometry. All esds (except the esd in the dihedral angle between two l.s. planes) are estimated using the full covariance matrix. The cell esds are taken into account individually in the estimation of esds in distances, angles and torsion angles; correlations between esds in cell parameters are only used when they are defined by crystal symmetry. An approximate (isotropic) treatment of cell esds is used for estimating esds involving l.s. planes.

### 2-(Bromomethyl)-3-methyl-1-(phenylsulfonyl)-1H-indole (I) Fractional atomic coordinates and isotropic or equivalent isotropic displacement parameters (Å^2^)

**Table:**

	*x*	*y*	*z*	*U*_iso_*/*U*_eq_	
C1	0.3530 (3)	0.4783 (2)	0.58255 (14)	0.0345 (5)	
C2	0.3824 (3)	0.3757 (2)	0.62843 (16)	0.0439 (6)	
H2	0.446932	0.378932	0.677377	0.053*	
C3	0.3113 (4)	0.2684 (2)	0.59810 (18)	0.0513 (7)	
H3	0.328360	0.198413	0.627406	0.062*	
C4	0.2149 (4)	0.2635 (3)	0.5247 (2)	0.0570 (7)	
H4	0.168902	0.190393	0.505784	0.068*	
C5	0.1868 (4)	0.3654 (3)	0.47978 (18)	0.0526 (7)	
H5	0.122660	0.361549	0.430761	0.063*	
C6	0.2562 (3)	0.4750 (2)	0.50893 (14)	0.0387 (5)	
C7	0.2515 (3)	0.5952 (2)	0.47726 (15)	0.0428 (6)	
C8	0.3405 (3)	0.6691 (2)	0.53034 (14)	0.0398 (5)	
C9	0.2921 (3)	0.6242 (2)	0.73345 (14)	0.0385 (5)	
C10	0.2971 (4)	0.5343 (3)	0.78836 (17)	0.0537 (7)	
H10	0.395387	0.489561	0.803309	0.064*	
C11	0.1513 (5)	0.5123 (3)	0.8207 (2)	0.0656 (9)	
H11	0.151977	0.452309	0.857811	0.079*	
C12	0.0065 (4)	0.5786 (3)	0.7982 (2)	0.0620 (8)	
H12	−0.090984	0.562026	0.819260	0.074*	
C13	0.0053 (4)	0.6692 (3)	0.74490 (18)	0.0585 (8)	
H13	−0.092489	0.714723	0.730838	0.070*	
C14	0.1477 (3)	0.6933 (3)	0.71194 (15)	0.0482 (6)	
H14	0.146765	0.754810	0.675899	0.058*	
C15	0.3845 (4)	0.7966 (2)	0.51928 (19)	0.0540 (7)	
H15A	0.397273	0.808599	0.465716	0.065*	
H15B	0.493150	0.813659	0.550710	0.065*	
C16	0.1665 (5)	0.6285 (3)	0.39759 (17)	0.0642 (9)	
H16A	0.177941	0.713609	0.389959	0.096*	
H16B	0.048275	0.607924	0.391753	0.096*	
H16C	0.218751	0.585444	0.360103	0.096*	
N1	0.4082 (3)	0.59886 (17)	0.59670 (12)	0.0369 (4)	
O1	0.5039 (3)	0.77321 (19)	0.68162 (13)	0.0614 (6)	
O2	0.6017 (2)	0.5675 (2)	0.72121 (13)	0.0596 (6)	
S1	0.47047 (8)	0.64724 (6)	0.68698 (4)	0.04217 (16)	
Br1	0.21361 (5)	0.91337 (3)	0.54636 (2)	0.07195 (15)	

### 2-(Bromomethyl)-3-methyl-1-(phenylsulfonyl)-1H-indole (I) Atomic displacement parameters (Å^2^)

**Table:**

	*U*^11^	*U*^22^	*U*^33^	*U*^12^	*U*^13^	*U*^23^
C1	0.0357 (11)	0.0336 (11)	0.0354 (12)	−0.0001 (9)	0.0087 (9)	−0.0030 (9)
C2	0.0492 (14)	0.0417 (13)	0.0413 (13)	0.0019 (11)	0.0085 (11)	0.0015 (11)
C3	0.0574 (17)	0.0350 (13)	0.0632 (18)	0.0002 (12)	0.0148 (14)	0.0037 (12)
C4	0.0605 (18)	0.0385 (14)	0.071 (2)	−0.0082 (13)	0.0083 (15)	−0.0132 (14)
C5	0.0523 (16)	0.0507 (16)	0.0517 (16)	0.0003 (13)	−0.0014 (13)	−0.0157 (13)
C6	0.0379 (12)	0.0400 (13)	0.0385 (13)	0.0043 (10)	0.0069 (10)	−0.0064 (10)
C7	0.0480 (14)	0.0442 (14)	0.0369 (12)	0.0126 (11)	0.0086 (11)	−0.0006 (11)
C8	0.0486 (14)	0.0343 (12)	0.0395 (13)	0.0082 (10)	0.0162 (11)	0.0029 (10)
C9	0.0442 (13)	0.0389 (12)	0.0319 (11)	−0.0030 (10)	0.0042 (10)	−0.0091 (10)
C10	0.0686 (19)	0.0485 (16)	0.0460 (15)	0.0103 (14)	0.0153 (14)	0.0029 (13)
C11	0.092 (3)	0.0532 (18)	0.0582 (19)	−0.0030 (17)	0.0335 (18)	0.0052 (15)
C12	0.0607 (19)	0.074 (2)	0.0563 (18)	−0.0052 (16)	0.0254 (15)	−0.0081 (16)
C13	0.0460 (15)	0.077 (2)	0.0536 (17)	0.0048 (15)	0.0101 (13)	−0.0018 (16)
C14	0.0479 (14)	0.0577 (17)	0.0382 (13)	0.0023 (13)	0.0042 (11)	0.0022 (12)
C15	0.0698 (19)	0.0387 (14)	0.0599 (18)	0.0037 (13)	0.0303 (15)	0.0048 (12)
C16	0.079 (2)	0.070 (2)	0.0418 (15)	0.0249 (18)	0.0027 (14)	0.0042 (15)
N1	0.0430 (11)	0.0327 (10)	0.0356 (10)	−0.0025 (8)	0.0082 (8)	−0.0037 (8)
O1	0.0733 (14)	0.0497 (12)	0.0623 (13)	−0.0260 (11)	0.0137 (11)	−0.0174 (10)
O2	0.0384 (10)	0.0789 (15)	0.0577 (12)	0.0025 (10)	−0.0047 (9)	−0.0067 (11)
S1	0.0380 (3)	0.0465 (4)	0.0413 (3)	−0.0093 (3)	0.0036 (2)	−0.0100 (3)
Br1	0.1035 (3)	0.03893 (17)	0.0794 (3)	0.01814 (16)	0.0331 (2)	0.00174 (15)

### 2-(Bromomethyl)-3-methyl-1-(phenylsulfonyl)-1H-indole (I) Geometric parameters (Å, º)

**Table:**

C1—C2	1.393 (4)	C10—C11	1.394 (5)
C1—C6	1.397 (4)	C10—H10	0.9300
C1—N1	1.419 (3)	C11—C12	1.374 (5)
C2—C3	1.388 (4)	C11—H11	0.9300
C2—H2	0.9300	C12—C13	1.372 (5)
C3—C4	1.393 (5)	C12—H12	0.9300
C3—H3	0.9300	C13—C14	1.379 (4)
C4—C5	1.377 (5)	C13—H13	0.9300
C4—H4	0.9300	C14—H14	0.9300
C5—C6	1.399 (4)	C15—Br1	1.992 (3)
C5—H5	0.9300	C15—H15A	0.9700
C6—C7	1.444 (4)	C15—H15B	0.9700
C7—C8	1.355 (4)	C16—H16A	0.9600
C7—C16	1.501 (4)	C16—H16B	0.9600
C8—N1	1.434 (3)	C16—H16C	0.9600
C8—C15	1.478 (4)	N1—S1	1.672 (2)
C9—C10	1.384 (4)	O1—S1	1.429 (2)
C9—C14	1.386 (4)	O2—S1	1.430 (2)
C9—S1	1.766 (3)		
			
C2—C1—C6	122.0 (2)	C10—C11—H11	119.8
C2—C1—N1	130.6 (2)	C13—C12—C11	120.2 (3)
C6—C1—N1	107.3 (2)	C13—C12—H12	119.9
C3—C2—C1	117.3 (3)	C11—C12—H12	119.9
C3—C2—H2	121.4	C12—C13—C14	120.6 (3)
C1—C2—H2	121.4	C12—C13—H13	119.7
C2—C3—C4	121.4 (3)	C14—C13—H13	119.7
C2—C3—H3	119.3	C13—C14—C9	118.9 (3)
C4—C3—H3	119.3	C13—C14—H14	120.6
C5—C4—C3	120.9 (3)	C9—C14—H14	120.6
C5—C4—H4	119.6	C8—C15—Br1	113.97 (19)
C3—C4—H4	119.6	C8—C15—H15A	108.8
C4—C5—C6	119.1 (3)	Br1—C15—H15A	108.8
C4—C5—H5	120.5	C8—C15—H15B	108.8
C6—C5—H5	120.5	Br1—C15—H15B	108.8
C1—C6—C5	119.4 (2)	H15A—C15—H15B	107.7
C1—C6—C7	108.0 (2)	C7—C16—H16A	109.5
C5—C6—C7	132.7 (2)	C7—C16—H16B	109.5
C8—C7—C6	108.4 (2)	H16A—C16—H16B	109.5
C8—C7—C16	127.0 (3)	C7—C16—H16C	109.5
C6—C7—C16	124.5 (3)	H16A—C16—H16C	109.5
C7—C8—N1	108.6 (2)	H16B—C16—H16C	109.5
C7—C8—C15	126.8 (3)	C1—N1—C8	107.7 (2)
N1—C8—C15	124.0 (3)	C1—N1—S1	120.44 (16)
C10—C9—C14	121.4 (3)	C8—N1—S1	127.74 (17)
C10—C9—S1	119.2 (2)	O1—S1—O2	120.11 (14)
C14—C9—S1	119.4 (2)	O1—S1—N1	106.42 (12)
C9—C10—C11	118.4 (3)	O2—S1—N1	106.72 (12)
C9—C10—H10	120.8	O1—S1—C9	110.12 (13)
C11—C10—H10	120.8	O2—S1—C9	107.87 (14)
C12—C11—C10	120.5 (3)	N1—S1—C9	104.46 (12)
C12—C11—H11	119.8		
			
C6—C1—C2—C3	0.0 (4)	C10—C9—C14—C13	−1.6 (4)
N1—C1—C2—C3	−179.5 (2)	S1—C9—C14—C13	175.7 (2)
C1—C2—C3—C4	0.2 (4)	C7—C8—C15—Br1	90.8 (3)
C2—C3—C4—C5	−0.1 (5)	N1—C8—C15—Br1	−98.9 (3)
C3—C4—C5—C6	−0.2 (5)	C2—C1—N1—C8	−179.5 (2)
C2—C1—C6—C5	−0.3 (4)	C6—C1—N1—C8	0.8 (3)
N1—C1—C6—C5	179.3 (2)	C2—C1—N1—S1	−20.7 (4)
C2—C1—C6—C7	−179.7 (2)	C6—C1—N1—S1	159.66 (17)
N1—C1—C6—C7	−0.1 (3)	C7—C8—N1—C1	−1.3 (3)
C4—C5—C6—C1	0.4 (4)	C15—C8—N1—C1	−173.2 (2)
C4—C5—C6—C7	179.6 (3)	C7—C8—N1—S1	−158.14 (19)
C1—C6—C7—C8	−0.8 (3)	C15—C8—N1—S1	30.0 (3)
C5—C6—C7—C8	179.9 (3)	C1—N1—S1—O1	−175.28 (19)
C1—C6—C7—C16	177.2 (3)	C8—N1—S1—O1	−21.1 (2)
C5—C6—C7—C16	−2.1 (5)	C1—N1—S1—O2	55.3 (2)
C6—C7—C8—N1	1.3 (3)	C8—N1—S1—O2	−150.5 (2)
C16—C7—C8—N1	−176.6 (3)	C1—N1—S1—C9	−58.8 (2)
C6—C7—C8—C15	172.8 (2)	C8—N1—S1—C9	95.4 (2)
C16—C7—C8—C15	−5.0 (5)	C10—C9—S1—O1	−136.9 (2)
C14—C9—C10—C11	1.5 (4)	C14—C9—S1—O1	45.7 (2)
S1—C9—C10—C11	−175.9 (2)	C10—C9—S1—O2	−4.1 (3)
C9—C10—C11—C12	0.1 (5)	C14—C9—S1—O2	178.5 (2)
C10—C11—C12—C13	−1.5 (5)	C10—C9—S1—N1	109.2 (2)
C11—C12—C13—C14	1.4 (5)	C14—C9—S1—N1	−68.2 (2)
C12—C13—C14—C9	0.2 (5)		

### 2-(Bromomethyl)-3-methyl-1-(phenylsulfonyl)-1H-indole (I) Hydrogen-bond geometry (Å, º)

**Table:**

*D*—H···*A*	*D*—H	H···*A*	*D*···*A*	*D*—H···*A*
C2—H2···O2	0.93	2.49	3.057 (4)	120
C2—H2···O1^i^	0.93	2.71	3.503 (4)	144
C10—H10···O1^i^	0.93	2.53	3.306 (4)	141
C12—H12···O2^ii^	0.93	2.77	3.302 (5)	118
C13—H13···O2^ii^	0.93	2.92	3.376 (4)	112
C16—H16*C*···O2^iii^	0.96	2.76	3.702 (4)	168
C4—H4···Br1^iv^	0.93	3.16	3.905 (4)	138
C12—H12···Br1^v^	0.93	3.16	3.922 (4)	141
C14—H14···Br1	0.93	2.99	3.894 (4)	165
C16—H16*A*···*Cg*(C9–C14)^vi^	0.96	2.97	3.765 (4)	142
C16—H16*B*···*Cg*(C1–C6)^vii^	0.96	2.97	3.823 (5)	149

Symmetry codes: (i) −*x*+1, *y*−1/2, −*z*+3/2; (ii) *x*−1, *y*, *z*; (iii) −*x*+1, −*y*+1, −*z*+1; (iv) *x*, *y*−1, *z*; (v) −*x*, *y*−1/2, −*z*+3/2; (vi) *x*, −*y*+3/2, *z*−1/2; (vii) −*x*, −*y*+1, −*z*+1.

### 2-[(E)-2-(2-Bromo-5-methoxyphenyl)ethenyl]-3-methyl-1-(phenylsulfonyl)-1H-indole (II) Crystal data

**Table:**

C_24_H_20_BrNO_3_S	*F*(000) = 984
*M**_r_* = 482.38	*D*_x_ = 1.489 Mg m^−^^3^
Monoclinic, *P*2_1_/*c*	Mo *K*α radiation, λ = 0.71073 Å
*a* = 8.4252 (9) Å	Cell parameters from 52480 reflections
*b* = 28.669 (3) Å	θ = 1.4–25.0°
*c* = 8.9462 (11) Å	µ = 2.03 mm^−^^1^
β = 95.445 (4)°	*T* = 298 K
*V* = 2151.1 (4) Å^3^	Solid, yellow
*Z* = 4	0.25 × 0.20 × 0.13 mm

### 2-[(E)-2-(2-Bromo-5-methoxyphenyl)ethenyl]-3-methyl-1-(phenylsulfonyl)-1H-indole (II) Data collection

**Table:**

Bruker D8 Venture Diffractometer	3905 reflections with *I* > 2σ(*I*)
Radiation source: micro focus sealed tube	*R*_int_ = 0.077
ω and φ scans	θ_max_ = 28.3°, θ_min_ = 3.3°
Absorption correction: multi-scan (*SADABS*; Krause *et al.*, 2015)	*h* = −11→11
*T*_min_ = 0.555, *T*_max_ = 0.745	*k* = −38→38
52479 measured reflections	*l* = −11→11
5296 independent reflections	

### 2-[(E)-2-(2-Bromo-5-methoxyphenyl)ethenyl]-3-methyl-1-(phenylsulfonyl)-1H-indole (II) Refinement

**Table:**

Refinement on *F*^2^	Primary atom site location: structure-invariant direct methods
Least-squares matrix: full	Secondary atom site location: difference Fourier map
*R*[*F*^2^ > 2σ(*F*^2^)] = 0.039	Hydrogen site location: inferred from neighbouring sites
*wR*(*F*^2^) = 0.099	H-atom parameters constrained
*S* = 1.03	*w* = 1/[σ^2^(*F*_o_^2^) + (0.0367*P*)^2^ + 1.0393*P*] where *P* = (*F*_o_^2^ + 2*F*_c_^2^)/3
5296 reflections	(Δ/σ)_max_ = 0.001
272 parameters	Δρ_max_ = 0.39 e Å^−^^3^
0 restraints	Δρ_min_ = −0.68 e Å^−^^3^

### 2-[(E)-2-(2-Bromo-5-methoxyphenyl)ethenyl]-3-methyl-1-(phenylsulfonyl)-1H-indole (II) Special details

**Table:**

Geometry. All esds (except the esd in the dihedral angle between two l.s. planes) are estimated using the full covariance matrix. The cell esds are taken into account individually in the estimation of esds in distances, angles and torsion angles; correlations between esds in cell parameters are only used when they are defined by crystal symmetry. An approximate (isotropic) treatment of cell esds is used for estimating esds involving l.s. planes.

### 2-[(E)-2-(2-Bromo-5-methoxyphenyl)ethenyl]-3-methyl-1-(phenylsulfonyl)-1H-indole (II) Fractional atomic coordinates and isotropic or equivalent isotropic displacement parameters (Å^2^)

**Table:**

	*x*	*y*	*z*	*U*_iso_*/*U*_eq_	
C1	0.6561 (3)	0.48550 (8)	0.3470 (2)	0.0469 (5)	
C2	0.6281 (3)	0.50829 (10)	0.2093 (3)	0.0614 (6)	
H2	0.568729	0.494387	0.128332	0.074*	
C3	0.6923 (4)	0.55236 (11)	0.1981 (3)	0.0717 (8)	
H3	0.676266	0.568262	0.107279	0.086*	
C4	0.7791 (4)	0.57337 (10)	0.3175 (4)	0.0742 (8)	
H4	0.820363	0.603085	0.305664	0.089*	
C5	0.8062 (3)	0.55132 (9)	0.4542 (3)	0.0643 (7)	
H5	0.863821	0.565906	0.534811	0.077*	
C6	0.7446 (3)	0.50632 (8)	0.4686 (2)	0.0473 (5)	
C7	0.7513 (3)	0.47458 (8)	0.5935 (2)	0.0461 (5)	
C8	0.6716 (2)	0.43516 (7)	0.5475 (2)	0.0421 (4)	
C9	0.7837 (3)	0.38292 (8)	0.2424 (3)	0.0489 (5)	
C10	0.8684 (3)	0.35109 (10)	0.3337 (3)	0.0673 (7)	
H10	0.820424	0.335580	0.408902	0.081*	
C11	1.0261 (4)	0.34260 (13)	0.3116 (4)	0.0860 (10)	
H11	1.084868	0.321232	0.372357	0.103*	
C12	1.0964 (4)	0.36561 (13)	0.2001 (5)	0.0861 (10)	
H12	1.202336	0.359691	0.185423	0.103*	
C13	1.0103 (4)	0.39724 (12)	0.1108 (4)	0.0805 (9)	
H13	1.058787	0.412993	0.036429	0.097*	
C14	0.8539 (4)	0.40589 (10)	0.1299 (3)	0.0643 (7)	
H14	0.795426	0.426986	0.067855	0.077*	
C15	0.6383 (3)	0.39387 (8)	0.6346 (2)	0.0444 (5)	
H15	0.536056	0.381323	0.623013	0.053*	
C16	0.7478 (3)	0.37332 (7)	0.7300 (2)	0.0438 (5)	
H16	0.851979	0.384173	0.732095	0.053*	
C17	0.7170 (2)	0.33490 (7)	0.8319 (2)	0.0400 (4)	
C18	0.5683 (3)	0.32918 (7)	0.8844 (2)	0.0438 (5)	
H18	0.486448	0.349562	0.851587	0.053*	
C19	0.5381 (3)	0.29388 (8)	0.9845 (3)	0.0490 (5)	
C20	0.6587 (3)	0.26380 (9)	1.0366 (3)	0.0599 (6)	
H20	0.639903	0.240384	1.104648	0.072*	
C21	0.8074 (3)	0.26891 (8)	0.9866 (3)	0.0580 (6)	
H21	0.889518	0.248965	1.022176	0.070*	
C22	0.8361 (3)	0.30307 (7)	0.8848 (3)	0.0455 (5)	
C23	0.8223 (4)	0.48672 (10)	0.7485 (3)	0.0681 (7)	
H23A	0.791508	0.463736	0.818252	0.102*	
H23B	0.784469	0.516817	0.776049	0.102*	
H23C	0.936409	0.487396	0.750554	0.102*	
C24	0.3489 (4)	0.25800 (13)	1.1295 (4)	0.0971 (12)	
H24A	0.238662	0.260695	1.147390	0.146*	
H24B	0.368142	0.227541	1.090527	0.146*	
H24C	0.414581	0.262561	1.222022	0.146*	
N1	0.6044 (2)	0.44108 (6)	0.39484 (19)	0.0449 (4)	
O1	0.5205 (2)	0.35818 (6)	0.3525 (2)	0.0635 (5)	
O2	0.5020 (2)	0.41358 (7)	0.1409 (2)	0.0722 (5)	
O3	0.3861 (2)	0.29237 (7)	1.0239 (2)	0.0704 (5)	
S1	0.58589 (7)	0.39595 (2)	0.27518 (6)	0.05060 (15)	
Br1	1.04254 (3)	0.30608 (2)	0.81730 (3)	0.06603 (11)	

### 2-[(E)-2-(2-Bromo-5-methoxyphenyl)ethenyl]-3-methyl-1-(phenylsulfonyl)-1H-indole (II) Atomic displacement parameters (Å^2^)

**Table:**

	*U*^11^	*U*^22^	*U*^33^	*U*^12^	*U*^13^	*U*^23^
C1	0.0452 (11)	0.0509 (12)	0.0454 (12)	0.0121 (9)	0.0092 (9)	0.0073 (10)
C2	0.0653 (16)	0.0700 (16)	0.0493 (13)	0.0168 (13)	0.0076 (11)	0.0139 (12)
C3	0.0784 (19)	0.0731 (18)	0.0660 (17)	0.0233 (15)	0.0194 (15)	0.0311 (15)
C4	0.0778 (19)	0.0526 (15)	0.096 (2)	0.0088 (14)	0.0262 (17)	0.0275 (15)
C5	0.0702 (17)	0.0495 (14)	0.0740 (17)	−0.0018 (12)	0.0115 (14)	0.0052 (13)
C6	0.0507 (12)	0.0435 (11)	0.0487 (12)	0.0066 (9)	0.0099 (10)	0.0047 (10)
C7	0.0494 (12)	0.0470 (12)	0.0419 (11)	0.0026 (9)	0.0044 (9)	0.0013 (9)
C8	0.0423 (11)	0.0477 (11)	0.0367 (10)	0.0055 (9)	0.0060 (8)	0.0032 (9)
C9	0.0481 (12)	0.0526 (13)	0.0459 (12)	−0.0051 (10)	0.0036 (10)	−0.0127 (10)
C10	0.0614 (16)	0.0698 (17)	0.0714 (17)	0.0112 (13)	0.0100 (13)	−0.0006 (14)
C11	0.0666 (19)	0.090 (2)	0.100 (2)	0.0239 (17)	0.0004 (18)	−0.0126 (19)
C12	0.0518 (16)	0.096 (2)	0.112 (3)	−0.0075 (16)	0.0195 (18)	−0.048 (2)
C13	0.082 (2)	0.080 (2)	0.086 (2)	−0.0191 (17)	0.0400 (18)	−0.0266 (18)
C14	0.0738 (17)	0.0673 (16)	0.0539 (14)	−0.0036 (13)	0.0167 (13)	−0.0091 (12)
C15	0.0440 (11)	0.0474 (12)	0.0425 (11)	−0.0010 (9)	0.0072 (9)	0.0013 (9)
C16	0.0408 (11)	0.0435 (11)	0.0477 (12)	0.0008 (9)	0.0069 (9)	−0.0008 (9)
C17	0.0424 (10)	0.0366 (10)	0.0399 (10)	0.0003 (8)	−0.0020 (8)	−0.0027 (8)
C18	0.0431 (11)	0.0431 (11)	0.0441 (11)	0.0017 (9)	−0.0021 (9)	0.0038 (9)
C19	0.0469 (12)	0.0514 (12)	0.0471 (12)	−0.0059 (10)	−0.0028 (10)	0.0076 (10)
C20	0.0614 (15)	0.0531 (14)	0.0631 (15)	−0.0030 (11)	−0.0060 (12)	0.0201 (12)
C21	0.0559 (14)	0.0472 (13)	0.0683 (16)	0.0097 (11)	−0.0081 (12)	0.0115 (12)
C22	0.0422 (11)	0.0425 (11)	0.0505 (12)	0.0036 (9)	−0.0022 (9)	−0.0057 (9)
C23	0.093 (2)	0.0577 (15)	0.0510 (14)	−0.0049 (14)	−0.0060 (14)	−0.0038 (12)
C24	0.079 (2)	0.110 (3)	0.106 (3)	−0.0124 (19)	0.0235 (19)	0.053 (2)
N1	0.0454 (9)	0.0490 (10)	0.0399 (9)	0.0047 (8)	0.0018 (7)	0.0024 (8)
O1	0.0581 (10)	0.0660 (11)	0.0666 (11)	−0.0193 (8)	0.0066 (8)	−0.0049 (9)
O2	0.0665 (11)	0.0939 (14)	0.0512 (10)	0.0062 (10)	−0.0195 (8)	−0.0042 (9)
O3	0.0510 (10)	0.0845 (13)	0.0760 (12)	−0.0058 (9)	0.0077 (9)	0.0337 (10)
S1	0.0441 (3)	0.0620 (4)	0.0443 (3)	−0.0022 (3)	−0.0033 (2)	−0.0053 (3)
Br1	0.04735 (15)	0.06198 (17)	0.0897 (2)	0.01104 (11)	0.01150 (13)	−0.00450 (14)

### 2-[(E)-2-(2-Bromo-5-methoxyphenyl)ethenyl]-3-methyl-1-(phenylsulfonyl)-1H-indole (II) Geometric parameters (Å, º)

**Table:**

C1—C6	1.393 (3)	C14—H14	0.9300
C1—C2	1.395 (3)	C15—C16	1.333 (3)
C1—N1	1.425 (3)	C15—H15	0.9300
C2—C3	1.382 (4)	C16—C17	1.469 (3)
C2—H2	0.9300	C16—H16	0.9300
C3—C4	1.375 (4)	C17—C18	1.389 (3)
C3—H3	0.9300	C17—C22	1.405 (3)
C4—C5	1.376 (4)	C18—C19	1.391 (3)
C4—H4	0.9300	C18—H18	0.9300
C5—C6	1.401 (3)	C19—O3	1.360 (3)
C5—H5	0.9300	C19—C20	1.380 (3)
C6—C7	1.438 (3)	C20—C21	1.377 (4)
C7—C8	1.358 (3)	C20—H20	0.9300
C7—C23	1.498 (3)	C21—C22	1.374 (3)
C8—N1	1.438 (3)	C21—H21	0.9300
C8—C15	1.459 (3)	C22—Br1	1.896 (2)
C9—C10	1.378 (4)	C23—H23A	0.9600
C9—C14	1.382 (3)	C23—H23B	0.9600
C9—S1	1.759 (2)	C23—H23C	0.9600
C10—C11	1.383 (4)	C24—O3	1.421 (3)
C10—H10	0.9300	C24—H24A	0.9600
C11—C12	1.376 (5)	C24—H24B	0.9600
C11—H11	0.9300	C24—H24C	0.9600
C12—C13	1.370 (5)	N1—S1	1.6770 (19)
C12—H12	0.9300	O1—S1	1.4237 (19)
C13—C14	1.368 (4)	O2—S1	1.4269 (18)
C13—H13	0.9300		
			
C6—C1—C2	121.5 (2)	C8—C15—H15	118.5
C6—C1—N1	107.75 (18)	C15—C16—C17	125.2 (2)
C2—C1—N1	130.7 (2)	C15—C16—H16	117.4
C3—C2—C1	117.2 (3)	C17—C16—H16	117.4
C3—C2—H2	121.4	C18—C17—C22	116.61 (19)
C1—C2—H2	121.4	C18—C17—C16	121.10 (18)
C4—C3—C2	121.8 (2)	C22—C17—C16	122.26 (19)
C4—C3—H3	119.1	C17—C18—C19	122.0 (2)
C2—C3—H3	119.1	C17—C18—H18	119.0
C3—C4—C5	121.4 (3)	C19—C18—H18	119.0
C3—C4—H4	119.3	O3—C19—C20	125.0 (2)
C5—C4—H4	119.3	O3—C19—C18	115.1 (2)
C4—C5—C6	118.3 (3)	C20—C19—C18	119.9 (2)
C4—C5—H5	120.9	C21—C20—C19	119.2 (2)
C6—C5—H5	120.9	C21—C20—H20	120.4
C1—C6—C5	119.8 (2)	C19—C20—H20	120.4
C1—C6—C7	108.3 (2)	C22—C21—C20	120.9 (2)
C5—C6—C7	131.8 (2)	C22—C21—H21	119.5
C8—C7—C6	108.00 (19)	C20—C21—H21	119.5
C8—C7—C23	128.0 (2)	C21—C22—C17	121.4 (2)
C6—C7—C23	123.7 (2)	C21—C22—Br1	117.89 (17)
C7—C8—N1	109.18 (18)	C17—C22—Br1	120.70 (17)
C7—C8—C15	128.9 (2)	C7—C23—H23A	109.5
N1—C8—C15	121.62 (19)	C7—C23—H23B	109.5
C10—C9—C14	120.9 (2)	H23A—C23—H23B	109.5
C10—C9—S1	119.18 (19)	C7—C23—H23C	109.5
C14—C9—S1	119.9 (2)	H23A—C23—H23C	109.5
C9—C10—C11	118.8 (3)	H23B—C23—H23C	109.5
C9—C10—H10	120.6	O3—C24—H24A	109.5
C11—C10—H10	120.6	O3—C24—H24B	109.5
C12—C11—C10	120.3 (3)	H24A—C24—H24B	109.5
C12—C11—H11	119.8	O3—C24—H24C	109.5
C10—C11—H11	119.8	H24A—C24—H24C	109.5
C13—C12—C11	120.0 (3)	H24B—C24—H24C	109.5
C13—C12—H12	120.0	C1—N1—C8	106.63 (17)
C11—C12—H12	120.0	C1—N1—S1	120.69 (15)
C14—C13—C12	120.7 (3)	C8—N1—S1	121.31 (15)
C14—C13—H13	119.7	C19—O3—C24	117.8 (2)
C12—C13—H13	119.7	O1—S1—O2	119.67 (12)
C13—C14—C9	119.3 (3)	O1—S1—N1	107.06 (10)
C13—C14—H14	120.4	O2—S1—N1	105.82 (11)
C9—C14—H14	120.4	O1—S1—C9	109.61 (12)
C16—C15—C8	122.9 (2)	O2—S1—C9	109.65 (12)
C16—C15—H15	118.5	N1—S1—C9	103.78 (10)
			
C6—C1—C2—C3	0.2 (4)	C17—C18—C19—O3	−179.1 (2)
N1—C1—C2—C3	178.4 (2)	C17—C18—C19—C20	1.2 (4)
C1—C2—C3—C4	−0.6 (4)	O3—C19—C20—C21	179.4 (2)
C2—C3—C4—C5	0.0 (4)	C18—C19—C20—C21	−0.9 (4)
C3—C4—C5—C6	0.8 (4)	C19—C20—C21—C22	−0.7 (4)
C2—C1—C6—C5	0.6 (3)	C20—C21—C22—C17	2.1 (4)
N1—C1—C6—C5	−177.9 (2)	C20—C21—C22—Br1	−177.9 (2)
C2—C1—C6—C7	179.6 (2)	C18—C17—C22—C21	−1.8 (3)
N1—C1—C6—C7	1.0 (2)	C16—C17—C22—C21	176.4 (2)
C4—C5—C6—C1	−1.1 (4)	C18—C17—C22—Br1	178.23 (15)
C4—C5—C6—C7	−179.8 (2)	C16—C17—C22—Br1	−3.6 (3)
C1—C6—C7—C8	1.2 (3)	C6—C1—N1—C8	−2.7 (2)
C5—C6—C7—C8	180.0 (3)	C2—C1—N1—C8	178.9 (2)
C1—C6—C7—C23	−173.6 (2)	C6—C1—N1—S1	−146.59 (16)
C5—C6—C7—C23	5.2 (4)	C2—C1—N1—S1	35.0 (3)
C6—C7—C8—N1	−2.9 (2)	C7—C8—N1—C1	3.5 (2)
C23—C7—C8—N1	171.6 (2)	C15—C8—N1—C1	177.77 (19)
C6—C7—C8—C15	−176.6 (2)	C7—C8—N1—S1	147.12 (16)
C23—C7—C8—C15	−2.1 (4)	C15—C8—N1—S1	−38.6 (3)
C14—C9—C10—C11	0.4 (4)	C20—C19—O3—C24	1.6 (4)
S1—C9—C10—C11	−177.1 (2)	C18—C19—O3—C24	−178.1 (3)
C9—C10—C11—C12	−0.1 (5)	C1—N1—S1—O1	−175.43 (16)
C10—C11—C12—C13	0.3 (5)	C8—N1—S1—O1	45.94 (19)
C11—C12—C13—C14	−0.8 (5)	C1—N1—S1—O2	−46.74 (19)
C12—C13—C14—C9	1.1 (4)	C8—N1—S1—O2	174.62 (17)
C10—C9—C14—C13	−0.9 (4)	C1—N1—S1—C9	68.69 (18)
S1—C9—C14—C13	176.6 (2)	C8—N1—S1—C9	−69.95 (18)
C7—C8—C15—C16	−45.2 (3)	C10—C9—S1—O1	−23.7 (2)
N1—C8—C15—C16	141.7 (2)	C14—C9—S1—O1	158.74 (19)
C8—C15—C16—C17	173.14 (19)	C10—C9—S1—O2	−157.0 (2)
C15—C16—C17—C18	−27.4 (3)	C14—C9—S1—O2	25.5 (2)
C15—C16—C17—C22	154.5 (2)	C10—C9—S1—N1	90.3 (2)
C22—C17—C18—C19	0.2 (3)	C14—C9—S1—N1	−87.2 (2)
C16—C17—C18—C19	−178.1 (2)		

### 2-[(E)-2-(2-Bromo-5-methoxyphenyl)ethenyl]-3-methyl-1-(phenylsulfonyl)-1H-indole (II) Hydrogen-bond geometry (Å, º)

**Table:**

*D*—H···*A*	*D*—H	H···*A*	*D*···*A*	*D*—H···*A*
C2—H2···O2	0.93	2.39	2.958 (4)	119
C3—H3···O2^i^	0.93	2.61	3.448 (3)	150
C24—H24*A*···Br1^ii^	0.96	3.03	3.699 (3)	128
C5—H5···*Cg*(C9–C14)^iii^	0.93	3.12	4.047 (3)	173
C20—H20···*Cg*(C17–C22)^iv^	0.93	3.15	3.978 (2)	149
C23—H23*C*···*Cg*(C1–C6)^iii^	0.96	3.11	4.036 (4)	162

Symmetry codes: (i) −*x*+1, −*y*+1, −*z*; (ii) *x*−1, −*y*+1/2, *z*+1/2; (iii) −*x*+2, −*y*+1, −*z*+1; (iv) *x*, −*y*+1/2, *z*+1/2.

### 2-[(E)-2-(2-Bromophenyl)ethenyl]-3-methyl-1-(phenylsulfonyl)-1H-indole (III) Crystal data

**Table:**

C_23_H_18_BrNO_2_S	*F*(000) = 920
*M**_r_* = 452.35	*D*_x_ = 1.516 Mg m^−^^3^
Monoclinic, *P*2_1_/*n*	Mo *K*α radiation, λ = 0.71073 Å
*a* = 12.5530 (8) Å	Cell parameters from 50531 reflections
*b* = 8.3533 (5) Å	θ = 1.4–25.0°
*c* = 19.7698 (11) Å	µ = 2.20 mm^−^^1^
β = 107.078 (2)°	*T* = 298 K
*V* = 1981.6 (2) Å^3^	Prism, yellow
*Z* = 4	0.36 × 0.31 × 0.24 mm

### 2-[(E)-2-(2-Bromophenyl)ethenyl]-3-methyl-1-(phenylsulfonyl)-1H-indole (III) Data collection

**Table:**

Bruker D8 Venture Diffractometer	3516 reflections with *I* > 2σ(*I*)
Radiation source: micro focus sealed tube	*R*_int_ = 0.058
ω and φ scans	θ_max_ = 27.0°, θ_min_ = 2.7°
Absorption correction: multi-scan (*SADABS*; Krause *et al.*, 2015)	*h* = −16→16
*T*_min_ = 0.514, *T*_max_ = 0.745	*k* = −10→10
50531 measured reflections	*l* = −25→25
4293 independent reflections	

### 2-[(E)-2-(2-Bromophenyl)ethenyl]-3-methyl-1-(phenylsulfonyl)-1H-indole (III) Refinement

**Table:**

Refinement on *F*^2^	Primary atom site location: structure-invariant direct methods
Least-squares matrix: full	Secondary atom site location: difference Fourier map
*R*[*F*^2^ > 2σ(*F*^2^)] = 0.036	Hydrogen site location: inferred from neighbouring sites
*wR*(*F*^2^) = 0.087	H-atom parameters constrained
*S* = 1.10	*w* = 1/[σ^2^(*F*_o_^2^) + (0.0262*P*)^2^ + 1.371*P*] where *P* = (*F*_o_^2^ + 2*F*_c_^2^)/3
4293 reflections	(Δ/σ)_max_ < 0.001
254 parameters	Δρ_max_ = 0.37 e Å^−^^3^
0 restraints	Δρ_min_ = −0.32 e Å^−^^3^

### 2-[(E)-2-(2-Bromophenyl)ethenyl]-3-methyl-1-(phenylsulfonyl)-1H-indole (III) Special details

**Table:**

Geometry. All esds (except the esd in the dihedral angle between two l.s. planes) are estimated using the full covariance matrix. The cell esds are taken into account individually in the estimation of esds in distances, angles and torsion angles; correlations between esds in cell parameters are only used when they are defined by crystal symmetry. An approximate (isotropic) treatment of cell esds is used for estimating esds involving l.s. planes.

### 2-[(E)-2-(2-Bromophenyl)ethenyl]-3-methyl-1-(phenylsulfonyl)-1H-indole (III) Fractional atomic coordinates and isotropic or equivalent isotropic displacement parameters (Å^2^)

**Table:**

	*x*	*y*	*z*	*U*_iso_*/*U*_eq_	
C1	0.3845 (2)	0.7169 (3)	0.40613 (15)	0.0514 (6)	
C2	0.2805 (2)	0.6430 (4)	0.3796 (2)	0.0725 (9)	
H2	0.242636	0.642367	0.331512	0.087*	
C3	0.2367 (3)	0.5708 (4)	0.4288 (3)	0.0861 (12)	
H3	0.168029	0.519654	0.413097	0.103*	
C4	0.2916 (3)	0.5724 (4)	0.5001 (3)	0.0847 (11)	
H4	0.258724	0.524019	0.531342	0.102*	
C5	0.3941 (3)	0.6443 (4)	0.52611 (19)	0.0691 (9)	
H5	0.430809	0.644742	0.574390	0.083*	
C6	0.4420 (2)	0.7169 (3)	0.47795 (15)	0.0510 (6)	
C7	0.5465 (2)	0.7977 (3)	0.48800 (13)	0.0461 (6)	
C8	0.55284 (19)	0.8444 (3)	0.42348 (13)	0.0430 (5)	
C9	0.5022 (2)	0.5840 (3)	0.28233 (12)	0.0434 (5)	
C10	0.6151 (2)	0.5774 (3)	0.28776 (13)	0.0516 (6)	
H10	0.658022	0.670085	0.295131	0.062*	
C11	0.6624 (3)	0.4304 (4)	0.28198 (15)	0.0618 (7)	
H11	0.737661	0.423945	0.285032	0.074*	
C12	0.5984 (3)	0.2933 (3)	0.27172 (14)	0.0627 (8)	
H12	0.630690	0.194953	0.267756	0.075*	
C13	0.4875 (3)	0.3010 (3)	0.26731 (15)	0.0609 (7)	
H13	0.445370	0.207621	0.260770	0.073*	
C14	0.4379 (2)	0.4461 (3)	0.27251 (13)	0.0532 (6)	
H14	0.362668	0.451289	0.269471	0.064*	
C15	0.6425 (2)	0.9296 (3)	0.40580 (12)	0.0443 (5)	
H15	0.625141	1.018220	0.376123	0.053*	
C16	0.7487 (2)	0.8842 (3)	0.43088 (12)	0.0425 (5)	
H16	0.763379	0.793865	0.459639	0.051*	
C17	0.84434 (19)	0.9639 (3)	0.41719 (11)	0.0393 (5)	
C18	0.8460 (2)	1.1291 (3)	0.40596 (13)	0.0495 (6)	
H18	0.784735	1.190395	0.407169	0.059*	
C19	0.9364 (3)	1.2033 (3)	0.39313 (14)	0.0572 (7)	
H19	0.934596	1.312866	0.384518	0.069*	
C20	1.0296 (2)	1.1155 (3)	0.39303 (13)	0.0542 (7)	
H20	1.091088	1.166316	0.385527	0.065*	
C21	1.0315 (2)	0.9525 (3)	0.40404 (13)	0.0481 (6)	
H21	1.093843	0.892468	0.403982	0.058*	
C22	0.93913 (19)	0.8795 (3)	0.41520 (12)	0.0402 (5)	
C23	0.6286 (3)	0.8304 (4)	0.55857 (14)	0.0608 (7)	
H23A	0.670017	0.734729	0.575877	0.091*	
H23B	0.589586	0.863969	0.591254	0.091*	
H23C	0.678808	0.913412	0.553866	0.091*	
N1	0.45078 (16)	0.8027 (2)	0.37072 (11)	0.0471 (5)	
O1	0.50537 (19)	0.8925 (2)	0.26566 (10)	0.0640 (5)	
O2	0.32416 (17)	0.7603 (3)	0.24957 (11)	0.0761 (6)	
S1	0.44041 (6)	0.77168 (8)	0.28558 (3)	0.05103 (17)	
Br1	0.94200 (2)	0.65291 (3)	0.42493 (2)	0.06143 (11)	

### 2-[(E)-2-(2-Bromophenyl)ethenyl]-3-methyl-1-(phenylsulfonyl)-1H-indole (III) Atomic displacement parameters (Å^2^)

**Table:**

	*U*^11^	*U*^22^	*U*^33^	*U*^12^	*U*^13^	*U*^23^
C1	0.0422 (13)	0.0425 (13)	0.0753 (18)	0.0112 (11)	0.0262 (13)	−0.0021 (12)
C2	0.0454 (15)	0.0663 (19)	0.107 (3)	0.0057 (14)	0.0239 (16)	−0.0137 (18)
C3	0.0554 (19)	0.062 (2)	0.160 (4)	−0.0014 (16)	0.061 (2)	−0.016 (2)
C4	0.089 (3)	0.058 (2)	0.138 (3)	0.0023 (18)	0.081 (3)	−0.005 (2)
C5	0.083 (2)	0.0539 (17)	0.091 (2)	0.0117 (16)	0.0584 (19)	0.0018 (15)
C6	0.0539 (15)	0.0407 (13)	0.0676 (17)	0.0137 (11)	0.0325 (13)	−0.0012 (12)
C7	0.0487 (14)	0.0398 (12)	0.0535 (14)	0.0145 (10)	0.0210 (11)	−0.0012 (11)
C8	0.0400 (12)	0.0379 (12)	0.0511 (13)	0.0111 (10)	0.0132 (10)	−0.0019 (10)
C9	0.0505 (14)	0.0375 (12)	0.0391 (12)	0.0021 (10)	0.0084 (10)	0.0030 (10)
C10	0.0521 (15)	0.0474 (14)	0.0529 (14)	−0.0001 (12)	0.0119 (12)	−0.0011 (12)
C11	0.0604 (17)	0.0616 (18)	0.0611 (17)	0.0177 (14)	0.0144 (13)	0.0008 (14)
C12	0.089 (2)	0.0433 (14)	0.0501 (15)	0.0187 (15)	0.0120 (15)	0.0001 (12)
C13	0.082 (2)	0.0415 (14)	0.0544 (16)	−0.0062 (14)	0.0129 (14)	−0.0009 (12)
C14	0.0561 (15)	0.0474 (15)	0.0535 (15)	−0.0034 (12)	0.0121 (12)	0.0005 (12)
C15	0.0483 (13)	0.0368 (12)	0.0478 (13)	0.0047 (10)	0.0145 (11)	0.0008 (10)
C16	0.0476 (13)	0.0362 (12)	0.0466 (13)	0.0064 (10)	0.0184 (11)	0.0002 (10)
C17	0.0448 (12)	0.0352 (11)	0.0375 (11)	0.0030 (9)	0.0117 (10)	−0.0026 (9)
C18	0.0584 (15)	0.0349 (12)	0.0540 (14)	0.0069 (11)	0.0148 (12)	−0.0014 (10)
C19	0.0750 (19)	0.0367 (13)	0.0567 (16)	−0.0078 (13)	0.0143 (14)	0.0024 (12)
C20	0.0537 (15)	0.0590 (17)	0.0484 (14)	−0.0158 (13)	0.0125 (12)	0.0007 (12)
C21	0.0423 (13)	0.0546 (15)	0.0479 (13)	0.0003 (11)	0.0139 (11)	−0.0029 (11)
C22	0.0458 (12)	0.0322 (11)	0.0419 (12)	0.0032 (9)	0.0115 (10)	−0.0017 (9)
C23	0.0679 (18)	0.0654 (18)	0.0511 (15)	0.0178 (14)	0.0204 (13)	−0.0022 (13)
N1	0.0404 (11)	0.0441 (11)	0.0564 (12)	0.0075 (9)	0.0134 (9)	−0.0016 (9)
O1	0.0944 (15)	0.0394 (10)	0.0564 (11)	0.0035 (10)	0.0194 (10)	0.0085 (8)
O2	0.0572 (12)	0.0791 (15)	0.0734 (13)	0.0209 (11)	−0.0100 (10)	−0.0017 (11)
S1	0.0548 (4)	0.0413 (3)	0.0503 (3)	0.0116 (3)	0.0049 (3)	0.0037 (3)
Br1	0.05484 (17)	0.03621 (14)	0.0976 (2)	0.00739 (11)	0.02920 (15)	−0.00128 (13)

### 2-[(E)-2-(2-Bromophenyl)ethenyl]-3-methyl-1-(phenylsulfonyl)-1H-indole (III) Geometric parameters (Å, º)

**Table:**

C1—C6	1.391 (4)	C13—C14	1.380 (4)
C1—C2	1.399 (4)	C13—H13	0.9300
C1—N1	1.428 (3)	C14—H14	0.9300
C2—C3	1.388 (5)	C15—C16	1.333 (3)
C2—H2	0.9300	C15—H15	0.9300
C3—C4	1.374 (5)	C16—C17	1.466 (3)
C3—H3	0.9300	C16—H16	0.9300
C4—C5	1.376 (5)	C17—C22	1.394 (3)
C4—H4	0.9300	C17—C18	1.399 (3)
C5—C6	1.404 (4)	C18—C19	1.381 (4)
C5—H5	0.9300	C18—H18	0.9300
C6—C7	1.437 (4)	C19—C20	1.381 (4)
C7—C8	1.358 (3)	C19—H19	0.9300
C7—C23	1.496 (4)	C20—C21	1.378 (4)
C8—N1	1.437 (3)	C20—H20	0.9300
C8—C15	1.458 (3)	C21—C22	1.383 (3)
C9—C14	1.387 (4)	C21—H21	0.9300
C9—C10	1.390 (4)	C22—Br1	1.902 (2)
C9—S1	1.759 (2)	C23—H23A	0.9600
C10—C11	1.383 (4)	C23—H23B	0.9600
C10—H10	0.9300	C23—H23C	0.9600
C11—C12	1.379 (4)	N1—S1	1.670 (2)
C11—H11	0.9300	O1—S1	1.424 (2)
C12—C13	1.370 (4)	O2—S1	1.427 (2)
C12—H12	0.9300		
			
C6—C1—C2	122.0 (3)	C9—C14—H14	120.6
C6—C1—N1	107.3 (2)	C16—C15—C8	122.0 (2)
C2—C1—N1	130.7 (3)	C16—C15—H15	119.0
C3—C2—C1	116.5 (3)	C8—C15—H15	119.0
C3—C2—H2	121.7	C15—C16—C17	125.8 (2)
C1—C2—H2	121.7	C15—C16—H16	117.1
C4—C3—C2	122.1 (3)	C17—C16—H16	117.1
C4—C3—H3	118.9	C22—C17—C18	116.2 (2)
C2—C3—H3	118.9	C22—C17—C16	121.9 (2)
C3—C4—C5	121.3 (3)	C18—C17—C16	121.9 (2)
C3—C4—H4	119.3	C19—C18—C17	121.6 (2)
C5—C4—H4	119.3	C19—C18—H18	119.2
C4—C5—C6	118.3 (3)	C17—C18—H18	119.2
C4—C5—H5	120.8	C18—C19—C20	120.3 (2)
C6—C5—H5	120.8	C18—C19—H19	119.9
C1—C6—C5	119.6 (3)	C20—C19—H19	119.9
C1—C6—C7	108.7 (2)	C21—C20—C19	120.0 (3)
C5—C6—C7	131.7 (3)	C21—C20—H20	120.0
C8—C7—C6	107.9 (2)	C19—C20—H20	120.0
C8—C7—C23	127.5 (3)	C20—C21—C22	119.0 (2)
C6—C7—C23	124.4 (2)	C20—C21—H21	120.5
C7—C8—N1	109.1 (2)	C22—C21—H21	120.5
C7—C8—C15	128.9 (2)	C21—C22—C17	123.0 (2)
N1—C8—C15	122.0 (2)	C21—C22—Br1	117.57 (18)
C14—C9—C10	121.0 (2)	C17—C22—Br1	119.44 (17)
C14—C9—S1	120.0 (2)	C7—C23—H23A	109.5
C10—C9—S1	118.91 (19)	C7—C23—H23B	109.5
C11—C10—C9	118.8 (3)	H23A—C23—H23B	109.5
C11—C10—H10	120.6	C7—C23—H23C	109.5
C9—C10—H10	120.6	H23A—C23—H23C	109.5
C12—C11—C10	120.2 (3)	H23B—C23—H23C	109.5
C12—C11—H11	119.9	C1—N1—C8	106.8 (2)
C10—C11—H11	119.9	C1—N1—S1	122.15 (18)
C13—C12—C11	120.5 (3)	C8—N1—S1	123.93 (17)
C13—C12—H12	119.7	O1—S1—O2	119.60 (13)
C11—C12—H12	119.7	O1—S1—N1	106.70 (11)
C12—C13—C14	120.5 (3)	O2—S1—N1	106.19 (12)
C12—C13—H13	119.7	O1—S1—C9	109.10 (12)
C14—C13—H13	119.7	O2—S1—C9	108.67 (13)
C13—C14—C9	118.9 (3)	N1—S1—C9	105.70 (10)
C13—C14—H14	120.6		
			
C6—C1—C2—C3	0.6 (4)	C22—C17—C18—C19	0.5 (4)
N1—C1—C2—C3	−178.8 (3)	C16—C17—C18—C19	180.0 (2)
C1—C2—C3—C4	0.6 (5)	C17—C18—C19—C20	−1.8 (4)
C2—C3—C4—C5	−1.0 (5)	C18—C19—C20—C21	1.6 (4)
C3—C4—C5—C6	0.1 (5)	C19—C20—C21—C22	−0.1 (4)
C2—C1—C6—C5	−1.5 (4)	C20—C21—C22—C17	−1.3 (4)
N1—C1—C6—C5	178.1 (2)	C20—C21—C22—Br1	176.59 (19)
C2—C1—C6—C7	178.4 (2)	C18—C17—C22—C21	1.1 (3)
N1—C1—C6—C7	−2.0 (3)	C16—C17—C22—C21	−178.4 (2)
C4—C5—C6—C1	1.1 (4)	C18—C17—C22—Br1	−176.75 (17)
C4—C5—C6—C7	−178.8 (3)	C16—C17—C22—Br1	3.7 (3)
C1—C6—C7—C8	−0.6 (3)	C6—C1—N1—C8	3.7 (2)
C5—C6—C7—C8	179.3 (3)	C2—C1—N1—C8	−176.8 (3)
C1—C6—C7—C23	176.2 (2)	C6—C1—N1—S1	155.22 (17)
C5—C6—C7—C23	−3.8 (4)	C2—C1—N1—S1	−25.3 (4)
C6—C7—C8—N1	3.0 (3)	C7—C8—N1—C1	−4.2 (2)
C23—C7—C8—N1	−173.7 (2)	C15—C8—N1—C1	178.2 (2)
C6—C7—C8—C15	−179.6 (2)	C7—C8—N1—S1	−155.05 (17)
C23—C7—C8—C15	3.7 (4)	C15—C8—N1—S1	27.3 (3)
C14—C9—C10—C11	1.1 (4)	C1—N1—S1—O1	171.57 (19)
S1—C9—C10—C11	−177.1 (2)	C8—N1—S1—O1	−41.8 (2)
C9—C10—C11—C12	−0.6 (4)	C1—N1—S1—O2	42.9 (2)
C10—C11—C12—C13	−0.2 (4)	C8—N1—S1—O2	−170.45 (19)
C11—C12—C13—C14	0.5 (4)	C1—N1—S1—C9	−72.4 (2)
C12—C13—C14—C9	−0.1 (4)	C8—N1—S1—C9	74.2 (2)
C10—C9—C14—C13	−0.7 (4)	C14—C9—S1—O1	−155.3 (2)
S1—C9—C14—C13	177.4 (2)	C10—C9—S1—O1	22.9 (2)
C7—C8—C15—C16	48.7 (4)	C14—C9—S1—O2	−23.3 (2)
N1—C8—C15—C16	−134.1 (2)	C10—C9—S1—O2	154.9 (2)
C8—C15—C16—C17	−178.7 (2)	C14—C9—S1—N1	90.3 (2)
C15—C16—C17—C22	−149.4 (2)	C10—C9—S1—N1	−91.5 (2)
C15—C16—C17—C18	31.1 (4)		

### 2-[(E)-2-(2-Bromophenyl)ethenyl]-3-methyl-1-(phenylsulfonyl)-1H-indole (III) Hydrogen-bond geometry (Å, º)

**Table:**

*D*—H···*A*	*D*—H	H···*A*	*D*···*A*	*D*—H···*A*
C2—H2···O2	0.93	2.37	2.949 (5)	120
C13—H13···O1^i^	0.93	2.73	3.420 (3)	132
C14—H14···O2^ii^	0.93	2.77	3.547 (4)	142
C19—H19···Br1^iii^	0.93	2.94	3.805 (3)	155
C5—H5···*Cg*(C9–C14)^iv^	0.93	2.96	3.806 (4)	153
C23—H23*A*···*Cg*(C1–C6)^iv^	0.96	3.21	3.999 (3)	110
C23—H23*B*···*Cg*(N1/C1/C6–C8)^v^	0.96	3.12	3.561 (3)	149

Symmetry codes: (i) *x*, *y*−1, *z*; (ii) −*x*+1/2, *y*−1/2, −*z*+1/2; (iii) *x*, *y*+1, *z*; (iv) −*x*+1, −*y*+1, −*z*+1; (v) −*x*+1, −*y*+2, −*z*+1.

## References

[bb1] Allen, F. H. (1981). *Acta Cryst.* B**37**, 900–906.

[bb2] Allen, F. H., Kennard, O., Watson, D. G., Brammer, L., Orpen, A. G. & Taylor, R. (1987). *J. Chem. Soc. Perkin Trans. 2*, pp. S1–19.

[bb3] Bassindale, A. (1984). *The Third Dimension in Organic Chemistry*, ch. 1, p. 11. New York: John Wiley and Sons.

[bb4] Beddoes, R. L., Dalton, L., Joule, T. A., Mills, O. S., Street, J. D. & Watt, C. I. F. (1986). *J. Chem. Soc. Perkin Trans. 2*, pp. 787–797.

[bb6] Bouthenet, E., Oh, K. B., Park, S., Nagi, N. K., Lee, H. S. & Matthews, S. E. (2011). *Bioorg. Med. Chem. Lett.* **21**, 7142–7145. 10.1016/j.bmcl.2011.09.072 22001028

[bb7] Bruker (2016). *APEX2* and *SAINT*. Bruker AXS Inc., Madison, Wisconsin, USA.

[bb8] Farrugia, L. J. (2012). *J. Appl. Cryst.* **45**, 849–854.

[bb9] Gentry, C. L., Egleton, R. D., Gillespie, T., Abbruscato, T. J., Bechowski, H. B., Hruby, V. J. & Davis, T. P. (1999). *Peptides*, **20**, 1229–1238. 10.1016/s0196-9781(99)00127-8 10573295

[bb10] Groom, C. R., Bruno, I. J., Lightfoot, M. P. & Ward, S. C. (2016). *Acta Cryst.* B**72**, 171–179. 10.1107/S2052520616003954 PMC4822653 27048719

[bb11] Guerrero, Y., Singh, S. P., Mai, T., Murali, R. K., Tanikella, L., Zahedi, A., Kundra, V. & Anvari, B. (2017). *Appl. Mater. Interfaces*, **9**, 19601–19611. 10.1021/acsami.7b03373 28524652

[bb12] Gunasekaran, B., Sureshbabu, R., Mohanakrishnan, A. K., Chakkaravarthi, G. & Manivannan, V. (2009). *Acta Cryst.* E**65**, o2069. 10.1107/S1600536809029985 PMC2970018 21577492

[bb13] Jasinski, J. P., Rinderspacher, A. & Gribble, G. W. (2009). *J. Chem. Crystallogr.* **40**, 40–47.

[bb15] Krause, L., Herbst-Irmer, R., Sheldrick, G. M. & Stalke, D. (2015). *J. Appl. Cryst.* **48**, 3–10. 10.1107/S1600576714022985 PMC4453166 26089746

[bb16] Liu, H., Yin, J., Xing, E., Du, Y., Su, Y., Feng, Y. & Meng, S. (2021). *Dyes Pigments*, **190**, 109327.

[bb17] Mackenzie, C. F., Spackman, P. R., Jayatilaka, D. & Spackman, M. A. (2017). *IUCrJ*, **4**, 575–587. 10.1107/S205225251700848X PMC5600021 28932404

[bb18] Macrae, C. F., Sovago, I., Cottrell, S. J., Galek, P. T. A., McCabe, P., Pidcock, E., Platings, M., Shields, G. P., Stevens, J. S., Towler, M. & Wood, P. A. (2020). *J. Appl. Cryst.* **53**, 226–235. 10.1107/S1600576719014092 PMC6998782 32047413

[bb19] Madhan, S., NizamMohideen, M., Pavunkumar, V. & MohanaKrishnan, A. K. (2022). *Acta Cryst.* E**78**, 1198–1203.

[bb20] Madhan, S., NizamMohideen, M., Pavunkumar, V. & Mohana­Krishnan, A. K. (2023*a*). *Acta Cryst.* E**79**, 521–525. 10.1107/S2056989023003821 PMC10242738 37288467

[bb21] Madhan, S., NizamMohideen, M., Pavunkumar, V. & MohanaKrishnan, A. K. (2023*b*). *Acta Cryst.* E**79**, 741–746. 10.1107/S2056989023006096 PMC10439409 37601405

[bb22] Okabe, N. & Adachi, Y. (1998). *Acta Cryst.* C**54**, 386–387.

[bb23] Parkin, A., Barr, G., Dong, W., Gilmore, C. J., Jayatilaka, D., McKinnon, J. J., Spackman, M. A. & Wilson, C. C. (2007). *CrystEngComm*, **9**, 648–652.

[bb24] Piscopo, E., Diurno, M. V., Mazzoni, O. & Ciaccio, A. M. (1990). *Boll. Soc. Ital. Biol. Sper.* **66**, 1181–1186. 2100530

[bb25] Ramathilagam, C., Saravanan, V., Mohanakrishnan, A. K., Chakkaravarthi, G., Umarani, P. R. & Manivannan, V. (2011). *Acta Cryst.* E**67**, o632. 10.1107/S1600536811004685 PMC3051985 21522386

[bb26] Schollmeyer, D., Fischer, G. & Pindur, U. (1995). *Acta Cryst.* C**51**, 2572–2575.

[bb27] Semenova, O., Kobzev, D., Yazbak, F., Nakonechny, F., Kolosova, O., Tatarets, A., Gellerman, G. & Patsenker, L. (2021). *Dyes Pigments*, **195**, 109745.

[bb28] Sheldrick, G. M. (2008). *Acta Cryst.* A**64**, 112–122. 10.1107/S0108767307043930 18156677

[bb29] Sheldrick, G. M. (2015). *Acta Cryst.* C**71**, 3–8.

[bb31] Spackman, P. R., Turner, M. J., McKinnon, J. J., Wolff, S. K., Grimwood, D. J., Jayatilaka, D. & Spackman, M. A. (2021). *J. Appl. Cryst.* **54**, 1006–1011. 10.1107/S1600576721002910 PMC8202033 34188619

[bb32] Spek, A. L. (2020). *Acta Cryst.* E**76**, 1–11. 10.1107/S2056989019016244 PMC6944088 31921444

[bb33] Umadevi, M., Saravanan, V., Yamuna, R., Mohanakrishnan, A. K. & Chakkaravarthi, G. (2013). *Acta Cryst.* E**69**, o1802–o1803. 10.1107/S1600536813031413 PMC3885060 24454236

[bb34] Westrip, S. P. (2010). *J. Appl. Cryst.* **43**, 920–925.

[bb35] Williams, T. M., Ciccarone, T. M., MacTough, S. C., Rooney, C. S., Balani, S. K., Condra, J. H., Emini, E. A., Goldman, M. E., Greenlee, W. J., Kauffman, L. R., *et al.* (1993). *J. Med. Chem.* **36**, 1291–1294. 10.1021/jm00061a022 7683725

